# From Plant to Chemistry: Sources of Antinociceptive Non-Opioid Active Principles for Medicinal Chemistry and Drug Design

**DOI:** 10.3390/molecules29040815

**Published:** 2024-02-09

**Authors:** Rita Turnaturi, Silvia Piana, Salvatore Spoto, Giuliana Costanzo, Lorena Reina, Lorella Pasquinucci, Carmela Parenti

**Affiliations:** 1 Department of Drug and Health Sciences, Medicinal Chemistry Section, University of Catania, Viale A. Doria 6, 95125 Catania, Italy; rita.turnaturi@unict.it (R.T.); silviapiana@outlook.com (S.P.); 2 Department of Drug and Health Sciences, Pharmacology and Toxicology Section, University of Catania, Viale A. Doria 6, 95125 Catania, Italy; salvospoto12@icloud.com (S.S.); cparenti@unict.it (C.P.); 3 Department of Biomedical and Biotechnological Sciences, University of Catania, Via Santa Sofia 97, 95123 Catania, Italy; giuliana.costanzo93@gmail.com; 4 Postgraduate School of Clinical Pharmacology and Toxicology, University of Catania, Via Santa Sofia 97, 95123 Catania, Italy; lorena.reina@hotmail.it

**Keywords:** natural compounds, pain, analgesic, drug discovery

## Abstract

Pain is associated with many health problems and a reduced quality of life and has been a common reason for seeking medical attention. Several therapeutics are available on the market, although side effects, physical dependence, and abuse limit their use. As the process of pain transmission and modulation is regulated by different peripheral and central mechanisms and neurotransmitters, medicinal chemistry continues to study novel ligands and innovative approaches. Among them, natural products are known to be a rich source of lead compounds for drug discovery due to their chemical structural variety and different analgesic mechanisms. Numerous studies suggested that some chemicals from medicinal plants could be alternative options for pain relief and management. Previously, we conducted a literature search aimed at identifying natural products interacting either directly or indirectly with opioid receptors. In this review, instead, we have made an excursus including active ingredients derived from plants whose mechanism of action appears from the literature to be other than the modulation of the opioid system. These substances could, either by themselves or through synthetic and/or semi-synthetic derivatives, be investigated in order to improve their pharmacokinetic characteristics and could represent a valid alternative to the opioid approach to pain therapy. They could also be the basis for the study of new mechanisms of action in the approach to this complex and disabling pathology.

## 1. Introduction

Plants’ active principles are known for their positive effects on human diseases and have always represented a major contribution to pharmacotherapy. Natural products (NPs) indeed are characterized by a wide chemical diversity, which results in different drug-like properties and provide interesting scaffolds for drug development [1]. Successful results in this field are achieved through an interdisciplinary method, including ethnobotanical, pharmacological, phytochemical, and biotechnological knowledge [2]. Regarding analytical methods for natural compounds, identification in extracts to pharmacologic approaches for their activity evaluation, and different chemical and biological procedures are known to find natural scaffolds with potential for novel drug development [3,4,5]. Numerous currently approved drugs are derived from both unchanged and modified natural products [6]. Generally, natural products are subject to small structural modifications to develop novel analogs with improved pharmacological profiles, better drug delivery methods, and minimal toxicity effects [7].

Phlorizin, first isolated from apple tree bark in 1835, plays an important role as a dietary polyphenol that is able to regulate glucose homeostasis [8]. More specifically, in diabetic rats, phlorizin was shown to inhibit intestinal and renal glucose uptake via sodium-dependent glucose transporters (SGLTs), resulting in a reduction of hyperglycemia without altering insulin secretion. Nowadays, the interest in combined SGLT2/SGLT1 inhibitors is growing continuously, and these compounds are also thought to be applied with insulin in the therapy of type 1 diabetes [9].

Natural sources also have a prominent role, particularly in the field of anticancer agents, since they provide 60% of currently used agents [10]. Among them, *Catharanthus roseus* (L.) G.Don, alkaloids (vincristine, vinblastine, vinorelbine, vindesine, and vinflunine) are the earliest developed microtubule-targeting agents approved for clinical use as useful drugs in the treatment of hematological and lymphatic neoplasms with high therapeutic potential.

Because of the chemical structural variety and different analgesic mechanisms, active principles from medicinal plants could be alternative options for pain relief and management. Nowadays, pain represents an emerging public health problem [11]. Pain modulation and its perception are regulated by both peripheral and central mechanisms. The process of pain transmission indeed involves different neurotransmitters (i.e., L-glutamate, γ-aminobutyric acid), ion channels (sodium and calcium), neuropeptides (calcitonin gene-related peptide, substance P) [12], cyclooxygenase products, super-oxide-anion, nitric oxide, and prostaglandin, with a role in the pathogenesis of pain [13,14]. Several therapeutics for pain treatment are available on the market, but the side effects, physical dependence, and abuse limit their use. Thus, the analgesic effects of natural products are continuously tested in various models of pain by experimental studies.

Morphine, the most abundant opiate found in *Papaver somniferum* L. has traditionally been used as a painkiller to alleviate moderate to severe pain. When opioids bind to opioid receptors inside the CNS signal transduction, molecular and cellular changes in the pain-signaling neurons are prevented. Opioid drugs explain their function through the mu opioid receptor (MOR), delta opioid receptor (DOR), and kappa opioid receptor (KOR), with differences in potency and selectivity [15]. The medicinal chemistry development of modified morphine derivatives as analgesics is a classic example of the structural simplification or complication of a NP that has allowed for the development of compounds—some of which are currently used in clinical practice.

*Cannabis sativa* L. contains different cannabinoids that have potential therapeutic value in pain management. Other than the widely studied antinociceptive activity of tetrahydrocannabinol (THC) or cannabidiol (CBD), the antinociceptive effects of other cannabis components, such as cannabichromene (CBC) and cannabigerol (CBG), have also been revealed. In particular, cannabigerol can exert antinociceptive effects on multiple pain models by activating TRPV1 and desensitizing it to block the transmission of pain signals. In addition, CBG can further activate CB2R, but not CB1R, to stimulate the release of β-endorphin, which greatly promotes the antinociceptive effect [16].

To complete our previous research [17], we have, therefore, focused on the search for natural analgesic compounds and their undertaken structural modifications, including the active principles or plant extracts (in toto) that interacted with mechanisms other than the opioid system.

## 2. Method Section

This review has been written based on the internet sources MEDLINE-PubMed and EMBASE. Common search keywords were pain, natural products, and analgesic effects of natural products. Then, the most relevant articles were selected and considered for this study. The databases consist of studies conducted in the last ten years. The articles were then screened to analyze the involvement of natural products in the pain process. All electronic search titles, selected abstracts, and full-text articles were carefully read to verify the expedience criterion. After the removal of duplicates, citations were limited to animal studies, leading to the identification of thirty-one natural sources that were potentially useful in pain management. A systematic screening of the articles was performed according to the criteria of (a) any biological activity that was the effect of natural products or their active principles on nociception based on animal models and their antinociceptive mechanisms of action, and (b) plant material and chemical elucidation.

In Table 1, the natural plant source, active principles, and chemical class of compounds with tested antinociceptive activities are reported.

## 3. Active Antinociceptive Principles

### 3.1. Caffeoylquinic Acid

**Figure 1:**
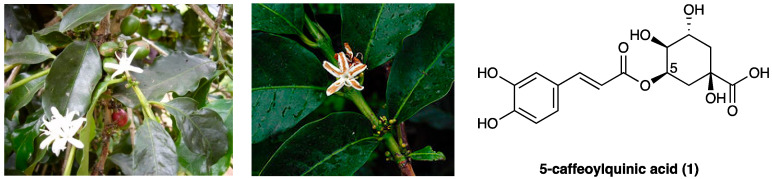
*Coffea arabica* L. and *Coffea canephora* Pierre ex A.Froehner and 5-caffeoylquinic acid chemical structure.

5-caffeoylquinic acid (**1**, 5-CQA) is the commonest individual chlorogenic acid (CGA) [52] found in plants, fruits, and vegetables, particularly in green coffee beans (*Coffea arabica* L. and *Coffea canephora* Pierre ex A.Froehner, Rubiaceae), but also in some types of berries (blueberries, blackberries, blackcurrants), apples, and pears [53]. It is a polyphenol formed by the esterification of caffeic acid with quinic acid (Figure 1) and makes up 70–80% of total CGAs. Due to its polyphenolic structure, 5-CQA has been demonstrated to have potent anti-inflammatory, antigenotoxic, and antioxidant properties, acting as a free-radical scavenger.

Despite the fact that 5-CQA is quite ineffective in acute pain, it presents anti-edematogenic and antinociceptive activities in animal models of carrageenan-induced inflammation and formalin-induced pain, respectively, with a mechanism of action that could be due to the inhibitory activity on peripheral TNF, NO, and several interleukins [54]. Bagdas et al. demonstrated that a single i.p. administration of 5-CQA (50, 100, and 200 mg/kg) produced a dose- and time-dependent antihyperalgesic effect comparable to gabapentin in CCI neuropathic pain; moreover, after 14 days an i.p. treatment at the dose of 100 mg/kg, attenuated mechanical hyperalgesia and ameliorated histopathological changes without affect motor function [55]. I.p. 5-CQA (100 mg/kg) chronic treatment (14 days) also exerted antihyperalgesic effects in a model of STZ-induced diabetic neuropathic pain [56]. In addition to the TNF- and NO-mediated mechanism of action, recent studies demonstrate that 5-CQA enhances K-selective voltage-gated channels (Kv) activities both in I_K,A_ and I_K,V_ channels in rat trigeminal ganglia neurons, gradually decreasing neuron excitability in trigeminal hyperalgesic conditions, such as in neuropathic and inflammatory pain [57,58].

A central mechanism of action has emerged in several studies. I.t. administered 5-CQA (at the dose of 0.5, 1, or 2 mg) had analgesic effects on mechanical, thermal, and cold hyperalgesia in a CCI rat model. This effect was significantly reversed by bicuculline, a GABA_A_ receptor antagonist. Conversely, strychnine (glycinergic antagonist), methysergide and ondansertron (serotoninergic antagonists), yohimbine (adrenergic antagonist), and naloxone (opioid antagonist) did not affect 5-CQA action, thus suggesting a GABA-mediated central mechanism of action. Additionally, i.p. or oral 5-CQA administration exerted anxiolytic and reduced brain damage in rats [59]. Finally, it has recently been demonstrated that 5-CQA forms a stable interaction with glutamate AMPA receptor at the GluA1 subunit, mainly through Van der Waals and electrostatic interactions. 5-CQA binding leads to GluA1 expression inhibition, reducing the abnormal AMPA receptor activation in neuropathic pain [60].

### 3.2. Puerarin

**Figure 2:**
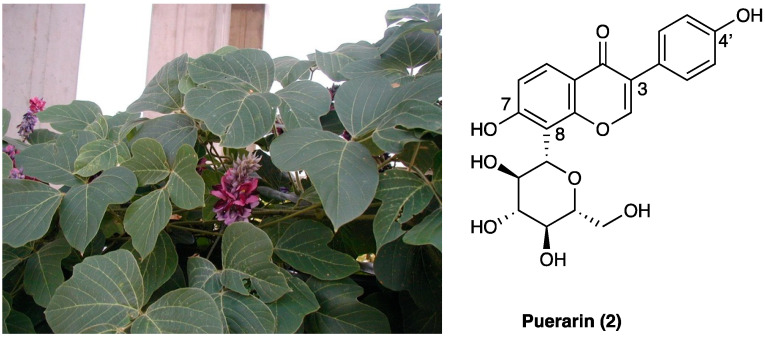
*Pueraria lobata* (Willd.) Ohwi. and its major constituent structure.

Puerarin (**2**, 8-*C*-glucoside of daidzein (4′,7-dihydroxy-isoflavon)) is one of the major isoflavonoids (a polyphenol sub-class derived by 3-phenyl-chromen-4-one skeletal structure) isolated from the root of the *Pueraria lobata* (Willd.) Ohwi. (Fabaceae) (Figure 2), also known as Gegen in traditional Chinese medicine, is an early medicinal herb frequently used to treat fever, diarrhea, emesis, cardiac dysfunctions, liver injury, weight loss, and toxicosis.

It is known that puerarin (**2**) inhibits calcium influx, improves microcirculation, reduces insulin resistance, scavenges ROS, and counteracts cell death. It is available in different dosage forms for clinical applications [136]. It has been extensively studied as an anti-inflammatory and antioxidant compound [137,138,139,140], and in recent years, its role in pain pathways has been investigated. In burn injury rats, puerarin (**2**) (100 mg/kg/day i.p., 30 min before burn for 3 days) decreased the up-regulated expression of P2X3 receptor protein and its mRNA in DRG neurons and reduced the primary afferent transmission on P2X3 receptor in DRG, leading to an improvement of thermal and mechanical hypersensitivity [141]. The same effect was observed in DRG of CCI rats after chronic i.p. treatment, confirming that puerarin (100 mg/kg/day i.p. for 14 days) can alleviate P2X3 nociceptive transmission in primary afferent neurons [142].

Puerarin’s (**2**) pain-relieving effect is not only due to ATP channel modulation but also to the interaction with other pain transmission mechanisms. Studies on paclitaxel-induced neuropathic pain in rats showed that local (i.pl.) injection of puerarin (**2**), dose-dependently, attenuated paclitaxel-induced mechanical allodynia and thermal hyperalgesia through DRG Na_v_ channels blockade. This effect was stronger in neuropathic animals than in the controls, suggesting a use-dependent blocking at the β1 subunit of the Na_v_1.8 channels [143]. In the same animal model, a single i.p. injection of puerarin (**2**) (10, 20, or 40 mg/kg) produced a short-term analgesic effect, meanwhile repeated doses of puerarin (**2**) (20 mg/kg, i.p. for consecutive 21 days) prevented the development of paclitaxel-induced neuropathic pain. This prophylactic effect of puerarin was associated with suppressed paclitaxel-induced transient TRPV1, and calcitonin gene-related peptide and substance P up-regulation in the dorsal root ganglia [144]. Concurrent effects on TRPV1 were also observed in a PSNL rat model, with a dose-dependent amelioration of mechanical allodynia and thermal hyperalgesia. In neuropathic pain models, i.t. puerarin (**2**) (4–100 nM for 7 days) had a dose-dependent effect comparable to fluorocitrate. It could inhibit spinal overexpression of cytokines and spinal glial activation, and up-regulate anti-inflammatory factors, suggesting a regulatory action on the neuroinflammatory process involved in neuropathic pain development [145,146]. Puerarin (**2**) alteration of TNF and IL-1β expression has been recently hypothesized to occur through TGF-β/Smad signaling activation. These results are corroborated by previous findings in spared nerve injury models, where intragastrically administered puerarin (**2**) was seen to act as a dual anti-depressant and analgesic agent comparable to ibuprofen and citalopram via ERK, CREB, and BDNF pathways [147].

### 3.3. Sinomenine

**Figure 3:**
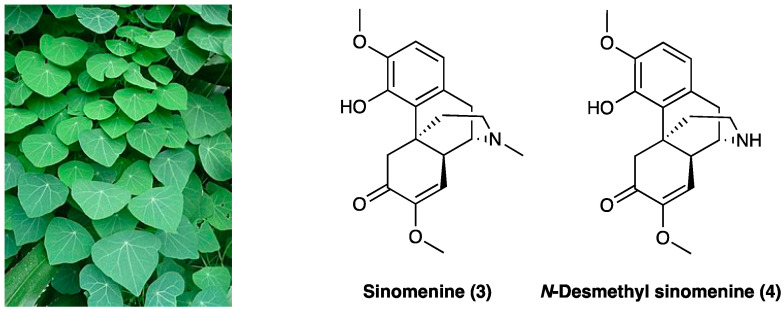
*Sinomenium acutum* Rehder & E.H. Wilson and its major alkaloids structures.

Sinomenine (**3**) is an alkaloid extracted from the roots of *Sinomenium acutum* Rehder & E.H. Wilson (Menispermaceae) (Figure 3), originally used in Japan and China to treat rheumatism.

Besides its antirheumatic activity, several studies have demonstrated sinomenine’s (**3**) efficacy in acute and chronic pain treatment during the last decade [177]. Sinomenine (**3**) is less unsafe if compared with opioids due to the absence of central inhibitory effects, which, albeit at a high dose (160 mg/kg, i.p.), could generate sedation and motor impairment. In rats, LD_50_ of sinomenine (**3**) is 535 or 580 mg/kg for i.p. or s.c. application, respectively. When administered chronically (150 mg/kg/day for 6 consecutive weeks), no irreversible organic damage was generated. In STZ-induced diabetic neuropathic pain, sinomenine (**3**) 30 mg/kg i.p. for 4 weeks) significantly decreased P2X3 receptor expression in DRG by reducing p38 MAPK activation and phosphorylation [178]. The amelioration of mechanical and thermal hyperalgesia was reversed by the co-treatment of P2X3-expressing engineered HEK293 cells with A317491, a P2X3-specific antagonist. Sinomenine (**3**) was found to reduce cellular excitability in small-sized DRG neurons via voltage-gated sodium channels [179]. In the SNL rat model, i.t. administered 20 mg/kg/for 3 or 28 days, sinomenine (**3**) alleviated apoptosis on DRG cells, confirmed in in vitro tests. It also down-regulated the expression of phosphorylation p38 MAPK, CREB, c-fos, CAMKII, NF-κB, COX-2, TLR4, IL-1β, and IL-17α in DRG cell and rat spinal cord tissue [180]. In the CCI rat model, chronically p.o. administered daily for 14 days at 20 mg/kg or 40 mg/kg, sinomenine (**3**) dose-dependently decreased thermal hyperalgesia and mechanical allodynia. It also modified the inflammatory response, reducing pro-inflammatory cytokines’ (such as TNF, IL-1β, and IL-6) mRNA expression [181]. In the same animal model, sinomenine (**3**) (two-week daily i.p treatment) was found able not only to reduce neuropathic pain symptoms but also to prevent the development of depressive-like behavior without tolerance. Sinomenine (**3**) effects were also blocked by the co-administration of bicuculline, a selective GABA_A_ receptor antagonist [182], showing a GABA_A_-mediated mechanism of action. Additionally, it was confirmed that the sinomenine (**3**) mechanism of action is independent of the opioid system since its activity was not reversed by concurrent naloxone administration and did not affect locomotor activity. Concordant findings were obtained with photochemically-induced spinal cord ischemic or sciatic nerve injury in rats and mice, with no apparent withdrawal symptoms following the end of i.p. or p.o. sinomenine (**3**) administration with gabapentin or ligustrazine hydrochloride [183,184]. Sinomenine (**3**), at less than ¼ of the effective dosage, potentiates gabapentin or ligustrazine hydrochloride, achieving the same antinociceptive efficacy through its neuroprotective anti-oxidant effects or also through GABA_A_ receptor activation, with minor adverse effects. The enhanced activity is believed to be pharmacodynamic and not pharmacokinetic because of the insignificant drug accumulation and the reduced active metabolite formation [185]. Also, the active metabolite, *N*-demethylsinomenine (**4**, Figure 3), showed an interesting activity in the neuropathic pain model [186].

In a study conducted by Zhou et al. [186], acute treatment with *N*-demethylsinomenine (**4**) (10–40 mg/kg, i.p.) attenuated, dose-dependently, mechanical allodynia both in CCI neuropathic pain and CFA inflammatory pain in mice, with a potency slightly higher than sinomenine. During the repeated treatment, *N*-demethylsinomenine (**4**) maintained its anti-allodynic effect without producing a carry-over effect. Pretreatment with bicuculline almost completely blocked *N*-demethylsinomenine (**4**) (40 mg/kg) anti-allodynia, both in CCI and CFA-treated mice.

**Figure 4:**
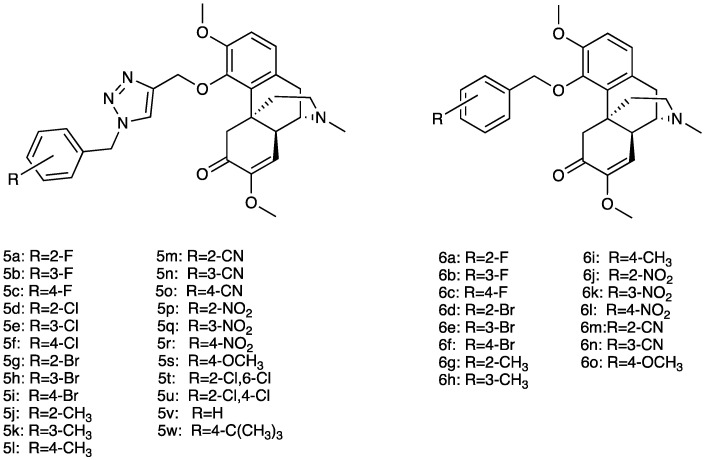
Structures of sinomenine derivatives.

Several sinomenine derivatives have been designed as anti-inflammatory agents, with NF-κB targeting for rheumatoid arthritis treatment (Figure 4). Compared with sinomenine, some of these derivatives showed improved activities, while others exhibited similar activity against NF-κB. In particular, the introduction of a substituted 1, 2, 3-triazole group with a proper length greatly enhanced the activity of these title compounds. In particular, compounds **6**a–w showed higher activity, while compounds **5**a–o showed similar activity against NF-κB [187].

### 3.4. Cedrol

**Figure 5:**
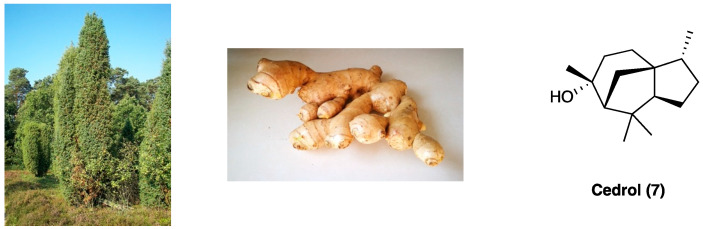
*Juniperus communis* L. and *Zengiber officinale* Roscoe and cedrol structure.

Cedrol (**7**, Figure 5) is a natural sesquiterpene with several biological activities, including antibacterial, sedative, anti-tumor, and regulation of the autonomic system [121]. It is mainly extracted from *Juniperus* species (Cupressaceae) and *Zingiber officinale* Roscoe (Zingiberaceae) (Figure 5) that, in traditional medicine, have been used to treat cystitis, arthritis, gout, and other inflammatory conditions. Essential oils obtained by steam distillation from the ripe, non-fermented cones of *Juniperus communis* L. are included in the European Pharmacopoeia [122]. Modern pharmacology has confirmed anti-obesity, antitumor, anti-ischemic, and anti-inflammation activity of *Z. officinale* Roscoe [123].

The anti-inflammatory and antioxidant effects have been considered responsible for cedrol action on neuropathic pain. In the CCI model, when i.p. administered at the dose of 40 mg/kg, cedrol alleviated mechanical and cold allodynia and heat hyperalgesia. Moreover, spinal TNF, IL-6, and MDA levels were lower, while thiol content was higher with respect to the treated group, highlighting its anti-inflammatory and antioxidant effect.

Cedrol has also been studied in rheumatoid pain. When p.o. administered at 20 mg/kg, it was found to inhibit chronic inflammation and pain in a dose-dependent manner with rapid onset and long duration, also ameliorating paw edema and arthritis score. The hypothesized mechanism of action was the inhibition of phosphorylated JAK3 protein through H-bond formation with ARG953 and ILE955 in the JAK3 active pocket. JAK3 pathway inhibition leads to the block of pro-inflammatory mediators’ secretion, therefore attenuating rheumatoid arthritis symptoms. It also decreased serum CFA, TNF, IL-1β, and MDA levels, while increasing thiol and SOD and GPx levels [124].

When re-crystalized using the cooling crystallization method with seven separate solvents, three crystal cedrol polymorphs emerged. They were separately evaluated in carrageenan-induced mouse paw edema, xylene-induced mouse ear edema, cotton pellet-induced mouse granuloma, and hot plate and acetic acid-induced mouse writhing. Form 1 emerged as the most effective pain relief at the oral dose of 20 mg/kg [125].

### 3.5. Genistein

**Figure 6:**
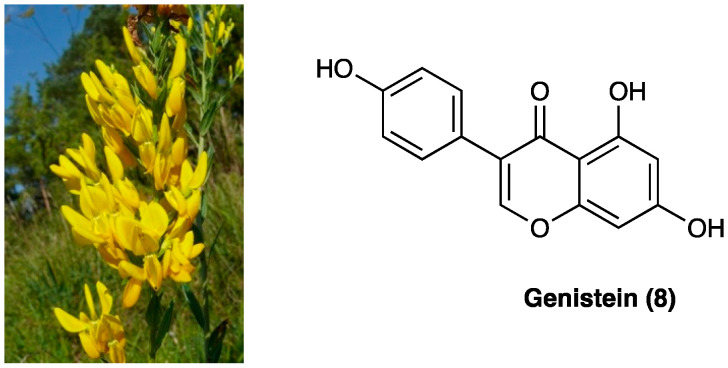
*Genista tinctoria* L. and the structure of genistein.

Genistein (**8**) is an isoflavone first isolated from *Genista tinctoria* L. (Fabaceae) (Figure 6), and widely distributed in fruits, leguminous plants, seeds, and vegetables such as barley meal, broccoli, caraway, cauliflower, clover seeds and sprouts, and sunflower. The major source of genistein is soybean, a cholesterol-free and high-protein legume. Naturally, isoflavones are usually associated with a glycosidic moiety; they are generally available as free aglycones only after food processing.

Preclinical studies have highlighted the antibacterial, antiviral, anti-inflammatory, antioxidant, estrogen- and angiotensin-like, and anti-cancer effects of genistein [188]. Its antinociceptive effect in chronic pain emerged first from a study by Shir et al., who observed that soy diets suppress the development of neuropathic pain behavior in rats undergoing PSNL injury [87]. In STZ diabetic neuropathy, chronic s.c. genistein administration (3 or 6 mg/kg daily, for three weeks, from the 2nd to the 5th week after STZ) relieved peripheral painful neuropathy, reverted pro-inflammatory cytokine and ROS overproduction, and restored NGF content in the sciatic nerve. Furthermore, it restored GSH content and GSH/GSSG ratio, improved antioxidant enzyme activities, and decreased ROS and lipoperoxide levels in the brain and liver. Finally, it restored iNOS and eNOS content and SOD activity in the thoracic aorta [88]. Genistein pain-relieving effects were also mediated by NF-κB, IL-1β, and IL-6 down-regulation both in peripheral and CNS. Studies on CCI-mouse and PNI-rat, such as sciatic nerve crush injury and complete sciatic nerve transection, have attributed genistein activity to several pathways [89,90]. The main target of genistein has been found to be ER, with a higher affinity for ERβ than ERα. Co-administration of a specific ERβ antagonist prevented both genistein anti-allodynic and anti-hyperalgesic action, whereas a specific ERα antagonist was ineffective [90]. ERβ receptor is highly expressed peripherally by immune cells and Schwann cells and centrally by neurons, microglia, and astrocytes. The concomitant administration of a non-selective ER antagonist reversed only the anti-allodynic effect, suggesting other pathways through which genistein exerted its effects, in particular, its anti-inflammatory and immunomodulatory effect. S.c. administered genistein has been shown to cross the BBB, so exerting its effect both peripherally and centrally. When chronically i.p administered in nerve-injured rats (once a day for 11 days, starting from the third day after surgeryat doses of 1, 3, 7.5, 15, and 30 mg/kg), it promoted nerve regeneration, proven by an enhancement in GAP-43 and MBP immunoreactivity. It also reduced IL-1β and TNF levels in injured nerve specimens, similar to gabapentin treatment. A significative motor improvement and paw-withdrawal threshold were also assessed, confirming the positive effect of chronic genistein in neuropathic pain [90]. Calcium voltage-gated channel Ca_v_3.3 has recently been found to be another target for genistein. Genistein directly blocks the activity of the human Ca_v_3.3 channel through polar interactions involving three hydroxyl groups and an aromatic interaction with the fused rings [91].

### 3.6. Solasodine

**Figure 7:**
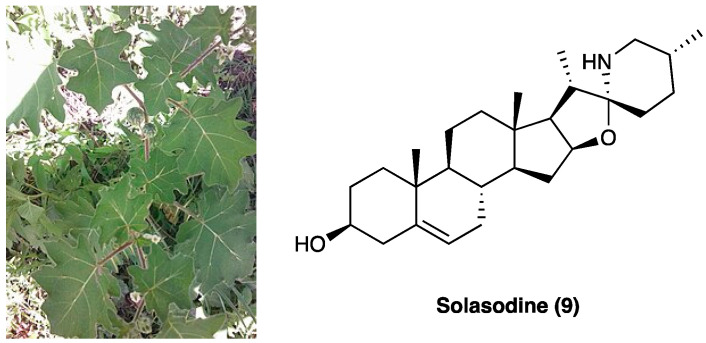
*Solanum virginianum* L. and the structure of solasodine.

Solasodine (**9**, Figure 7), one of the main components of the ethanolic extract of *Solanum virginianum* L. (Solanaceae), is commonly known as kantakari and is one of the active ingredients of the Ayurvedic medicine Dashamula, traditionally used to treat nerves and nourish muscle tone.

The entirely plant has been used for its antiasthmatic, anti-inflammatory, antinociceptive, antioxidant, and hepatoprotective activities. It has been demonstrated that p.o. administration of the ethanolic extract of S. virginianum L. dose-dependently attenuated CCI neuropathic pain [151]. An increase in paw withdrawal threshold was seen at both the tested doses, 100 and 200 mg/kg in cold and hot plate test, confirming a reduction in thermal allodynia comparable to the standard drug pregabalin (10 mg/kg). The antinociceptive effect was also assessed through Von Frey’s filaments and Randal–Selitto test. Ethanolic extract did not impair motor performance. The pain-relieving effect has been associated with solasodine (Figure 7), a steroidal glycoalkaloid with potential anticonvulsant [152], antinociceptive [153], and neuroprotective effects [154]. In vitro analyses also showed that CCI-induced reductions in SOD, catalase, and GSH levels were significantly refurbished by solasodine, while MDA and nitrite levels were decreased concomitantly to IL-1β and TNF levels. The interaction with Ca_v_ and Na_v_1.7 channels was hypothesized as two possible mechanisms of action. Molecular docking studies have assessed the binding to the Ca_v_ 2.2 and 2.3 channels and their inhibition through competition with Ca^2+^/N-lobe, but not to the Na_v_1.7 channel.

### 3.7. Sanguinarine

**Figure 8:**
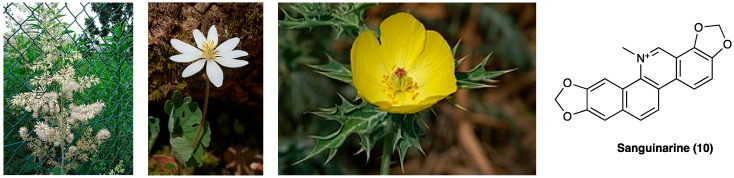
*Macleaya cordata* (Willd.) R.Br., *Sanguinaria canadensis* L., *Argemone mexicana* L. and structure of sanguinarine.

Sanguinarine is a benzyl isoquinoline alkaloid (**10**, Figure 8) extracted from the root of Papaveraceae plants, whose pharmacological activities such as anti-hypertensive, anti-microbial, anti-platelet, anti-trypanosome, anti-tumor, and anti-osteoclast formation have been assessed. It is the main active ingredient of *Macleaya cordata* (Willd.) R.Br. (Papaveraceae) extract, and of *Sanguinaria canadensis* L. roots (Papaveraceae) and *Argemone mexicana* L. seeds (Papaveraceae).

Previous studies have found sanguinarine as the main component (51.6%) of the alkaloid-rich fraction extract of the aerial parts of *Fumaria officinalis* L. (Papaveraceae), widely used in inflammatory and painful conditions [126]. Sanguinarine is a toxic polycyclic ammonium ion with no capability to penetrate the BBB. Sanguinarine anti-inflammatory activity has already been assessed [127], and its effects on neuropathic pain have been evaluated only recently. In CCI rats, i.p. sanguinarine (1.00, 2.50, and 6.25 mg/kg from the day of surgery every three days) increased the mechanical withdrawal threshold and thermal withdrawal latency. In addition, it inhibited the activation of microglia and decreased the expression of phosphorylated-p38 and TNF, IL-1β, and IL-6 in CCI spinal dorsal horn in a dose-dependent manner, thus, identifying microglia activation and p38 MAPK pathway as sanguinarine routes of action [128]. Inhibition of phosphorylated-p30 and NF-κB p65 were also assessed [129]. A recent study assessed sanguinarine as a full selective agonist of the TRPA1 channel (EC_50_ 0.09 mM), causing an increase in intracellular calcium levels and up-regulation of mouse dorsal root ganglion excitability [130]. Mutagenesis studies have confirmed that Cys421, Cys621, Cys641, Cys665, and Lys710 mediate the TRPA1 activation elicited by sanguinarine synergistically. In particular, Cys621 may play a central role because the current was abolished completely by C621A mutation, while the others were still functional with dramatically reduced currents.

### 3.8. (−)-Cassine

**Figure 9:**
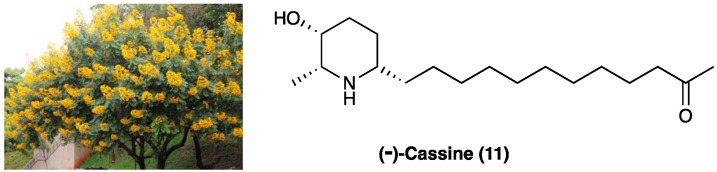
The structure of (−) cassine and the arborous species *Senna spectabilis* (DC.) Irwin & Barneby by which it is extracted.

(−)-Cassine (**11**) is a piperidine alkaloid isolated from flowers, fruits, and leaves of *Senna spectabilis* (DC.) Irwin & Barneby (synonym of *Cassia spectabilis,* Figure 9), an arborous species of Fabaceae. *S. spectabilis* (DC.) Irwin & Barneby has a traditional use as an anti-microbial, anti-tumoral, anti-ulcerogenic, laxative, analgesic, and anti-inflammatory traditional medicine.

Anti-inflammatory, antioxidant, and antinociceptive activity of (−)-cassine and (−)-spectaline (**59**), a co-metabolite of (−)-cassine, were evaluated [156]. The total synthesis of (−)-cassine has been reported via a diastereoselective Pd(II)-catalyzed cyclization strategy [157]. It has been evaluated in acute and chronic pain models, demonstrating that this active metabolite has systemic, spinal, and supraspinal anti-nociceptive properties. In CFA-induced chronic inflammation, (−)-cassine reduced mechanical hyperalgesia, with minor efficacy if compared to gabapentin.

When p.o. administered at different doses (3, 30, or 60 mg/kg) on the 5th day after neuropathic pain induction in a mice model of PSNL, (−)-cassine markedly reduced mechanical hyperalgesia. Its analgesic effects have been linked to the inhibition of TRPV1 and TRPA1, down-regulation of COX-2, MAP/ERK pathway, and NF-κB expression in the spinal cord. TRPV1 and TRPA1 targeting has been demonstrated by inhibition of the licking induced by the TRPA1 agonist cinnamaldehyde and by attenuation of cold hyperalgesia tetrafluoroethane-induced.

### 3.9. Hautriwaic Acid

**Figure 10:**
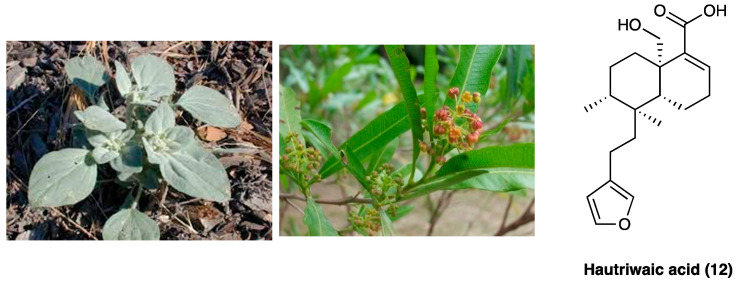
*Eremocarpus setigerus* (Hook.) Benth. and *Dodonaea viscosa* Jacq., and the structure of hautriwaic acid.

Hautriwaic acid (**12**) is extracted from *Eremocarpus setigerus* (Hook.) Benth. (Euphorbiaceae), also known as *Croton setigerus*, and *Dodonaea viscosa* Jacq. (Sapindaceae) (Figure 10), a medical plant traditionally used for its anti-diarrheal, antibacterial, analgesic, antiviral, antiulcer, antioxidant, anti-inflammatory, and gastroprotective activities.

*D. viscosa* Jacq., active principles are mainly terpenoids and flavonoids, among which the diterpene hautriwaic acid (**12**) has been demonstrated to have important anti-inflammatory and hepatoprotective activities [73]. This diterpene, isolated from dichloromethane extract, showed marked edema reduction (comparable to indomethacin) when topically (0.25, 0.5, and 1.0 mg/ear) or i.p. chronically (15 mg/kg once daily for 10 days) administered in 12-O-tetradecanoylphorbol 13-acetate mice ear edema model [74]. In an experimental model of rheumatoid arthritis induced by kaolin/carrageenan injection in the right knee of mice, p.o. administered hautriwaic acid (**12**) was shown to have an immunomodulatory effect by reducing pro-inflammatory cytokines (IL-1β, IL-6, and TNF) levels and enhancing IL-10 activity, with an activity comparable or higher than diclofenac, used as a positive control. It has recently been demonstrated that hautriwaic acid exerts a positive effect in an experimental model of HIV-induced neuropathy, but not in paclitaxel-induced neuropathy, by inhibiting TTX-S sodium channels in small diameter DRG neurons.

### 3.10. Tanshinones and Phenolic Acids

**Figure 11:**
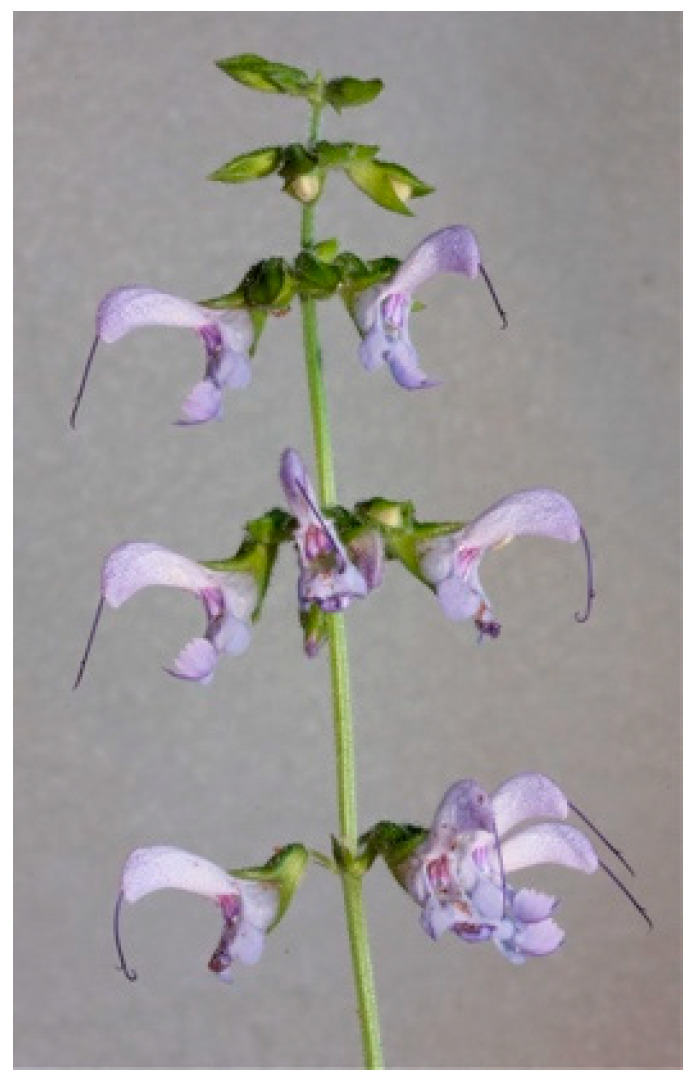
*Salvia miltiorrhiza* Bunge.

Dry root and rhizome of *Salvia miltiorrhiza* Bunge (Figure 11), a perennial herb belonging to le Labiatae family, are commonly known as Dan Shen, a medicament listed in the Chinese Pharmacopoeia traditionally used to treat cardiovascular diseases, chronic hepatic failure, and diabetes.

**Figure 12:**
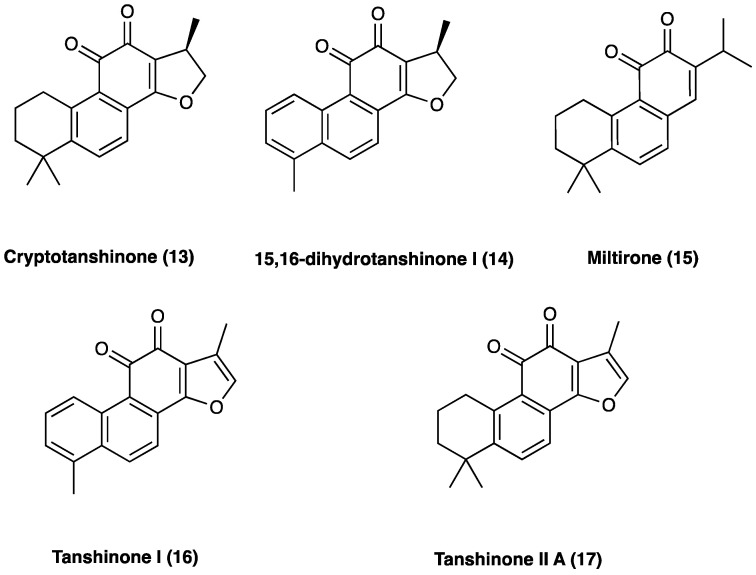
Structure of active tanshinones.

The main active ingredients of Dan Shen are diterpene tanshinones and phenolic acids. There have been more than 40 tanshinones isolated from Dan Shen, among which are cryptotanshinone (**13**), 15,16-dihydrotanshinone I (**14**), miltirone (**15**), tanshinone I (**16**), and tanshinone II A (**17**), that possess significant antioxidant, anti-inflammatory, and antineoplastic activities (Figure 12).

**Figure 13:**
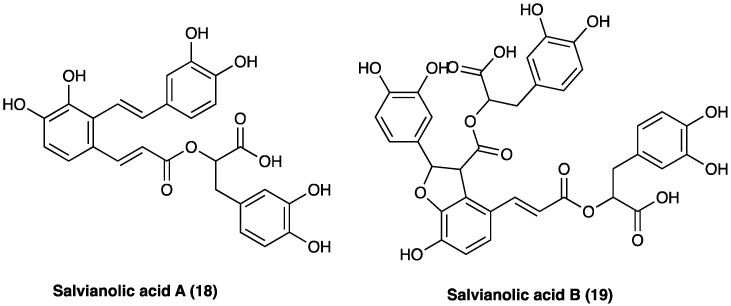
Structure of active phenolic acids.

Phenolic acids with antioxidant, anticoagulant, and cell protection activities [158] include caffeic acid monomers as well as oligomers, salvianolic acids, rosmarinic acid, and lithospermic acids. Salvianolic acid A (**18**) and B (**19**) are the major water-soluble constituents, and their content in *S. miltiorrhiza* Bunge is more abundant than in other Labiaceae species (Figure 13). *S. milthorizza* Bunge extract has been evaluated on a monosodium urate-induced pain mice model, showing its antinociceptive and anti-inflammatory activity mediated by the reduction in LPS-induced NO release [189].

In the last decade, several studies have also evaluated the potential analgesic properties of the association (mixture extract, ME) between *S. milthorriza* Bunge and *Agrimonia pilosa* Ledeb. extracts, and of some of the abovementioned active ingredients of Dan Shen individually. *A. pilosa* Ledeb., belonging to the Rosaceae family, is distributed over eastern Asia and eastern Europe. It has been used as a traditional medicinal herb to treat abdominal pain, sore throat, headaches, and parasitic infections. Several studies have shown *A. pilosa* Ledeb. antioxidant, antinociceptive, anti-inflammatory, and anti-allergic effects. *A. pilosa* Ledeb. has been reported to produce an anti-nociceptive effect in ICR strain mice in both tail-flick and hot-plate tests [190]. A study by Hwang et al. [191] evaluated several mixture ratios, defining the 1:1 ratio of a 50% EtOH extract of *A. pilosa* Ledeb. and an 80% EtOH extract of *S. milthorriza* Bunge., as the mixture extract with the most significant therapeutic potential for treating gout pain. In a mice osteoarthritis pain model, one-off and one-week p.o. treatment with ME reduced pain thresholds in a dose-dependent manner. ME also reduced plasma TNF, IL-6, and CRP levels. In LPS-stimulated RAW 264.7 cells, ME inhibited the release of NO, PGE2, LTB4, and IL-6, increased the phosphorylation of PPAR-γ protein, and downregulated TNF-α and MAPKs proteins expression in a concentration-dependent (from 1 to 100 μg/mL) manner. Furthermore, ME ameliorated the progression of ear edema in mice. Furthermore, repeated administration of ME for 7 days (once daily) showed a profound antinociceptive effect, suggesting that ME did not induce antinociceptive tolerance. In a mouse collagen-induced arthritis (CIA) model, both oral single and repeated treatments with ME decreased pain threshold, attenuated CRP, TNF, IL-6, COX-1, COX-2, and NF-κB levels in plasma and ankle tissue [192].

#### 3.10.1. Tanshinones: Cryptotanshinone

Cryptotanshinone (**13**, Figure 12) belongs to the tanshinones diterpenoid family, and is one of the main active compounds isolated from the root of *S. miltiorrhiza* Bunge. Recent studies have shown that cryptotanshinone effectively protects against cardiac dysfunction and plays a potent role in anti-tumorigenesis without affecting the survival of noncancerous cells. Furthermore, it ameliorates the symptoms of rheumatoid arthritis by suppressing proinflammatory cytokines in rats [193]. In a mouse oxaliplatin-induced neuropathic pain, a single cryptotanshinone administration (30 mg/kg p.o.) significantly reduced pain threshold without motor or neuronal alterations; 7 days repeated administrations of 10 mg/kg highlighted its effectiveness and potency. Cryptotanshinone was also found to selectively interfere with NF-κBp65 expression [194]. In CCI neuropathic pain, chronic p.o. cryptotanshinone administration suppressed the increase in IL-1β, IL-6, TNF, PI3K/Akt signaling, determining an overall improvement in the paw withdrawal mechanical threshold and thermal withdrawal latency.

#### 3.10.2. Tanshinones: Tanshinone IIA

Tanshinone II A (**17**, Figure 12) is one of the major diterpenes extracted from the roots of *S. milthorizza* Bunge. Several studies have exploited its analgesic activity in cancer-induced bone pain, inflammatory pain, pancreatitis-induced pain, and visceral pain. In rat STZ-induced neuropathic pain, tanshinone II A significantly improved mechanical allodynia and thermal hyperalgesia. Through electrophysiological and biochemical methods, it was elucidated that tanshinone II A normalized the altered activity of primary sensitive neurons by lowering the enhanced TTX-R and TTX-S sodium channel currents [195]. In mouse oxaliplatin-induced neuropathic pain, 7-day repeated p.o. tanshinone II A administration (10 mg/kg p.o.) significantly reduced pain thresholds. This compound was also found to be non-toxic since it did not induce motor or neurological impairment. Contextually, Dan Shen and its active constituents showed remarkable and selective inhibitory activities on glioblastoma cells lines LN-229 (IC_50_: 50.0, 48.2 and 51.9 μM respectively for Dan Shen standardized extract, tanshinone II A and cryptotanshinone), next to healthy but high proliferative cell lines enterocytes (IC_50_ > 250 μM for tanshinone II A and cryptotanshinone) and keratinocytes (IC_50_ > 100 and 97 μM respectively for tanshinone II A and cryptotanshinone), highlighting Dan Shen neuroprotective properties. Tanshinone II A selectively interfered with NF-κB p65 expression (with a specular and significant modulation of IKBα) in the CNS.

Tanshinone II A i.p. administration (10, 25, and 50 mg/kg) 30 min prior to and daily after operation for 14 days), dose-dependently, also inhibited SNL-induced mechanical hyperalgesia and, as revealed by OX42 levels, it effectively repressed the activation of spinal microglial activation. Meanwhile, tanshinone II A also decreased the expressions of inflammatory cytokines TNF and IL-1β in the spinal cord. Furthermore, tanshinone II A inhibited oxidative stress by significantly rescuing SOD activity and decreasing malondialdehyde. Moreover, tanshinone II A depressed SNL-induced MAPKs activation, thus acting as an immune response down regulator [196]. In the same rat pain model, tanshinone II A was also found to act through the JNK pathway and decreased MCP-1 release, dose dependently. Co-treatment of tanshinone II A and JNK inhibitor (SP600125), did not significantly increase mechanical PWT and MCP-1 expression compared with the tanshinone II A-treated group [197].

#### 3.10.3. Phenolic Acids: Salvianolic Acid A

Salvianolic acid A (**18**, Figure 13) is extracted from the aqueous faction of *S. milthorrizha* Bunge roots. It has antioxidant activity, inhibits hepatic fibrosis, liver injury, and thrombosis, protects the heart and brain from damage, and restores vascular reactivity in diabetic rats. When administered (1 and 3 mg/kg p.o.) in STZ-induced diabetic rats for 10 weeks after overt diabetes, salvianolic acid A increased peripheral blood perfusion and vascular activities, improved peripheral nerve function, and decreased vascular eNOS expression and blood glucose, lipid, von Willebrand factor (vWf), and malondialdehyde levels. The beneficial effects of salvianolic acid A on plantar microcirculation and peripheral nerve function in diabetic rats have been attributed to improvements in lipid and glucose metabolism, the inhibition of AGEs formation, and the development of oxidative stress-related nervous and vascular damage [198].

#### 3.10.4. Phenolic Acids: Salvianolic Acid B

Salvianolic acid B (**19**, Figure 13) is the enantiomer of salvianolic acid A, extracted from the aqueous faction of *Salvia milthorrizha* Bunge roots. It was evaluated in rat CCI-induced neuropathic pain, demonstrating its effectiveness in reducing mechanical hyperalgesia when administered i.p. at the dose of 100 mg/kg. Due to the poor chemical stability and bioavailability of salvianolic acid B, liposomes were developed as drug carriers for parental administration. According to in vivo studies, encapsulation, especially into PEGylated liposomes, increased and prolonged the antihyperalgesic activity [199].

### 3.11. Caffeic Acid Phenylethyl Ester

**Figure 14:**
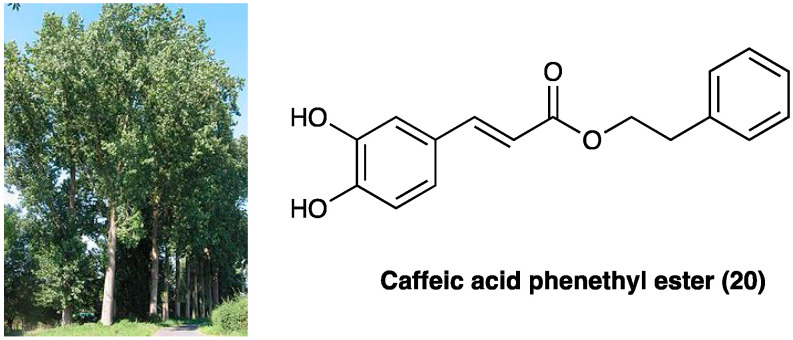
*Populus* × *canadensis* Moench and the structure of caffeic acid phenethyl ester.

Caffeic acid phenethyl ester (**20**, Figure 14) is the main ingredient of Chinese propolis (i.e., honeybees make propolis from resins of *Populus* × *canadensis* Moench, Salicaceae), which has been widely used in traditional Chinese medicine to treat various diseases [133]. It has antioxidative, antitumor, and anti-inflammatory effects. For instance, it was reported that caffeic acid phenethyl ester exerted therapeutic effects on atherosclerosis and Alzheimer’s disease [134]. Caffeic acid phenethyl ester also ameliorated LPS-induced microglial activation and motor incoordination and, at the dose of 25 mg/kg, i.p., for 7 days, relieved neuropathic pain behaviors induced by CCI in mice [135]. It also inhibited CCI-induced activation of microglia, suppressed the phosphorylation of p38 mitogen-activated protein kinase, inhibited the translocation of NF-κB and decreased the expression of proinflammatory cytokines tumor necrosis factor-α, IL-1β, and IL-6.

### 3.12. Fruticuline A

**Figure 15:**
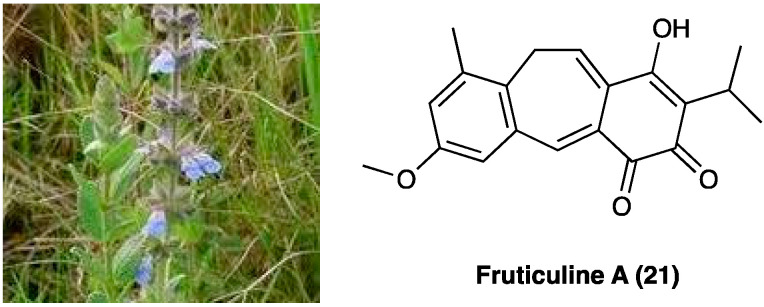
*Salvia lachnostachys* Benth and structure of fruticuline A.

*Salvia lachnostachys* Benth (Lamiaceae) is a herb native of Brazil. Its ethanolic extract from leaves (SLE) contains several triterpenes and diterpenes, among them, fruticuline A (**21**, Figure 15), a norabietane diterpenoid, emerged for its anti-inflammatory (paw edema and pleurisy induced by carrageenan injection) and antihyperalgesic effects [148,149].

Crude SLE contains about 3% of fruticuline A (**21**). In a study by Santos et al. SLE (100 mg/kg, p.o. route) was evaluated for its effects on SNI in rats [150]. The oral administration of SLE for up to 15 days significantly inhibited SNI-induced mechanical hyperalgesia and decreased immobility in the FST. In the formalin test, SLE and fruticuline A significantly reduced the frequency of paw licking during the first and second phases and decreased edema. SLE and fruticuline A did not alter the locomotor analysis (open field test without clonidine treatment), validating the absence of toxicity.

### 3.13. Gallic Acid

**Figure 16:**
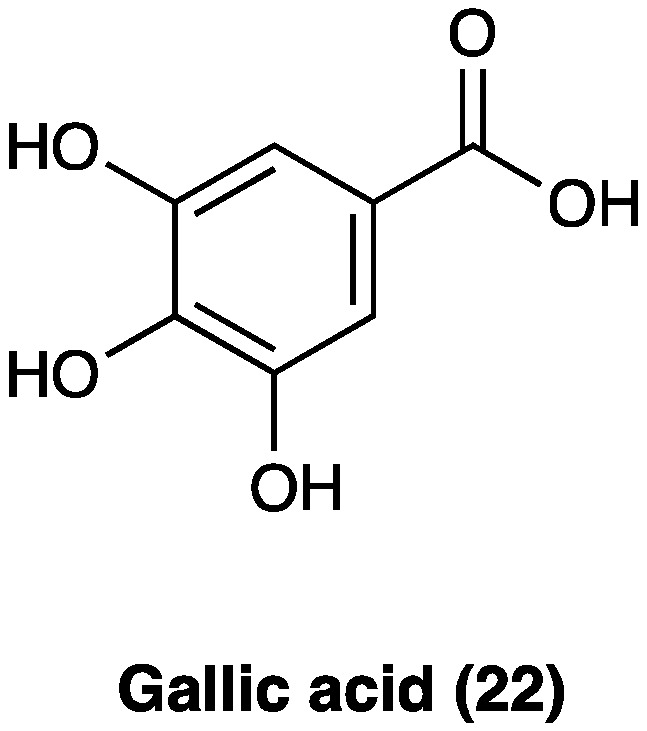
Structure of gallic acid.

Gallic acid (**22**, Figure 16) is a polyphenolic compound found in a wide variety of fruits, nuts, and plants, such as *Cornus officinalis* Torr. ex Dur. (Cornaceae), *Eucalyptus globulus* Labill. (Myrtaceae), gallnuts (*Quercus infectoria* G. Oliver, Fagaceae), rhubarb (*Rheum officinale* Baill., *Rheum palmatum* L., Polygonaceae), and sumac (*Rhus chinensis* Mill., Anacardaceae). It is also the principal antioxidant component of tea extract and several Ayurvedic herbs [61] and a component of Qufeng Zhitong capsules, a traditional Chinese medicine also clinically used for neuropathic pain [62].

Experimentally, gallic acid (**22**) has been documented to produce cardioprotective, anti-diabetic, anti-hyperlipidemic, anti-inflammatory, anti-depressive, and neuroprotective effects without any serious toxicity (no observable adverse effects at 5000 mg/kg per o.s.) [63,64,65,66]. Its analgesic properties have been widely studied in acute [67] as well as chronic and neuropathic pain models. In a study conducted by Kaur et al. [63] i.v. treatments with gallic acid (**22**) (20 and 40 mg/kg for 10 consecutive days) attenuated paclitaxel-induced pain behavior and related biochemical changes, suggesting gallic acid (**22**) ability to prevent neuronal firing, neurodegeneration, and neuroinflammation. Gallic acid (**22**) has been shown to inhibit histamine release, free radical scavenging action, oxidative stress, neuroinflammation and cytokine production, thermal and mechanical hyperalgesia and allodynia, tissue total calcium, TNF and the calcium influx TRPA1-mediated. Moreover, gallic acid (**22**), p.o. administered, decreased the spontaneous nociception triggered by allyl isothiocyanate, cinnamaldehyde, and H_2_O_2_. Carrageenan-induced allodynia and edema were largely reduced by the pretreatment with gallic acid (**22**). It was also capable of decreasing cold and mechanical allodynia in a CCI neuropathic pain model. It has been found that gallic acid (**22**) acts by inhibiting the NF-κB/STAT pathway through the P2X7 receptor. In a model of visceral pain, gallic acid (**22**) or P2X7 shRNA treatment could diminish spinal cord, DRG, and hippocampus P2X7 receptor expressions. Treatments with gallic acid (**22**) and P2X7 shRNA successfully increased the hippocampus BDNF level in comorbid rats, also indicating alleviated depression [68]. In CCI neuropathic pain, i.p. gallic acid (**22**) (100 mg/kg) for 1 week increased mechanical withdrawal threshold and thermal withdrawal latency accompanied by inhibition of the upregulated expression of P2X7 and TNF at both mRNA and protein levels and reduced NF-κB and phosphorylated-STAT3 in the dorsal root ganglia. At the same time, gallic acid (**22**) significantly decreased the co-expression of P2X7 and glial fibrillary acidic protein in the dorsal root ganglia. In addition, gallic acid (**22**) could suppress the ATP-activated current in HEK293 cells transfected with the plasmid expressing P2X7 but had no effect on the ATP activation current of P2X7-mutant plasmid (with the point mutation sequence of the key site where gallic acid (**22**) binds to the P2X7 receptor). The underlying molecular mechanisms have been addressed to the downregulation of P2X7 receptor expression, reduction of mature TACE release, inhibition of TNF-α expression, and suppression of the NF-κB/STAT3 signaling pathway [69].

### 3.14. Isosakuranetin

**Figure 17:**
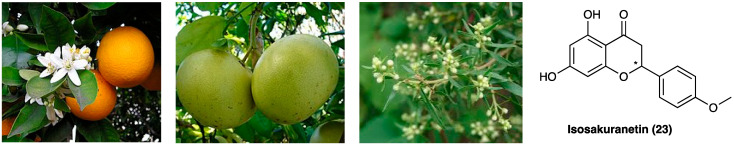
*Citrus sinensis* (L.) Osbeck, *Citrus paradisi* Macfad, *Baccharis dracunculifolia* DC., and the chemical structure of isosakuranetin.

Isosakuranetin (**23**, Figure 17) is a flavanone found in many plants such as citrus fruits (*Citrus sinensis* (L.) Osbeck and *Citrus paradisi* Macfad, Rutaceae) and Brazilian green propolis (*Baccharis dracunculifolia* DC. Asteraceae) [49], recently described as transient receptor potential melastatin 3 (TRPM3) blocker in in vitro studies [50]. When i.p administered, it elicited antinociceptive effects in mouse hot plate assay and in a chemical-induced inflammatory pain model.

In CCI, peripheral neuropathy i.p. isosakuranetin (1.5, 3, or 6 mg/kg) dose-dependently alleviated mechanical, thermal, and cold hyperalgesia in the von Frey test, Hargreaves’ plantar test, and cold plate test, respectively. In the rotarod test, at tested doses, isosakuranetin did not significantly affect motor performance, confirming the absence of toxicity [51].

### 3.15. Chlorogenic Acid

**Figure 18:**
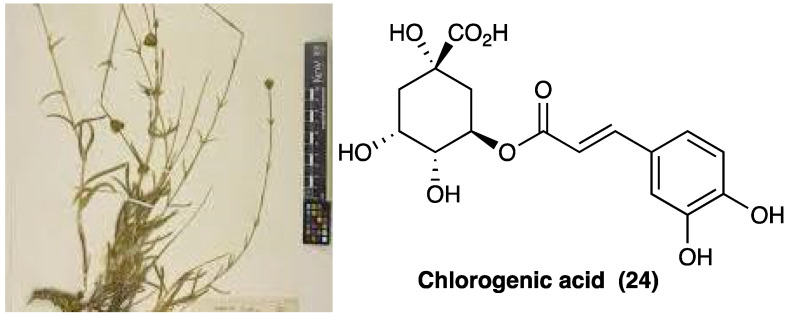
*Sideritis bilgeriana* P.H.Davis and chemical structure of chlorogenic acid.

*Sideritis bilgeriana* P.H.Davis belongs to the Lamiaceae family (Figure 18). The aerial parts of plants are used in teas and natural remedies to relieve colds, gastrointestinal symptoms, and inflammatory processes [174].

The methanolic extract of *S. bilgeriana* P.H.Davis has been evaluated through HPLC analyses, and chlorogenic acid (**24**, Figure 18) has been found to be its main active component. The methanolic extract of *S. bilgeriana* P.H.Davis activity was evaluated in several pain models, showing to reduce (at the doses of 50, 100, and 200 mg/kg, p.o.) mechanical hyperalgesia, MPO activity, and pain behavior. In the carrageenan-induced pleurisy, methanolic extract of *S. bilgeriana* P.H.Davis (100 mg/kg p.o. 60 min. before the injection of carrageenan) significantly reduced leukocyte (polymorphonuclear) count and TNF and IL-1β levels in the pleural cavity such as analysis of bone marrow showed a decrease in of pro-inflammatory IL-6 cytokine and NF-κB factor level. In the PSNL model, at the same dose, mechanical and thermal hyperalgesia were reduced on the first day and during the 7 days of evaluation. Moreover, the methanolic extract of *S. bilgeriana* P.H.Davis treatment produced no noticeable side effects, motor impairment, and gastric or hepatic injury.

### 3.16. Daturalactone, 12-Deoxywithastramonolide, and Daturilin

**Figure 19:**
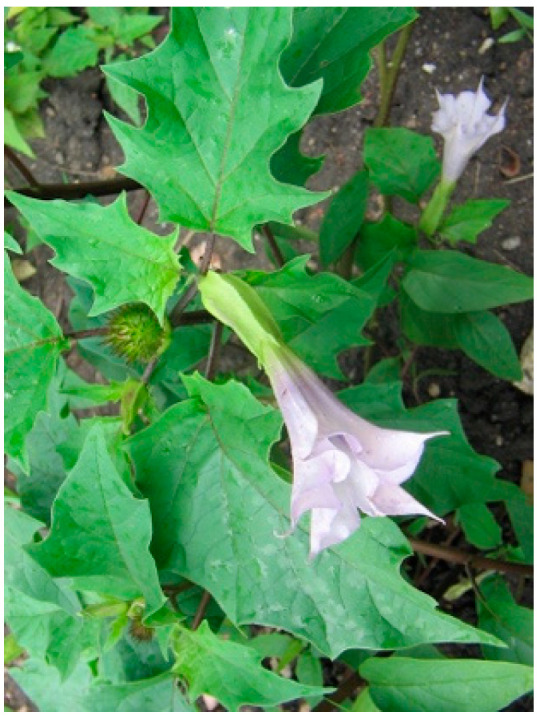
*Datura stramonium* L.

*Datura stramonium* L. (Solanaceae) (Figure 19) is a renowned medicinal herb from the Solanaceae family. In Ayurvedic medicine, *D. stramonium* L. is described as a useful remedy for various human illnesses [70]. Its pharmaceutical actions are antiasthmatic, anticancer, antimicrobial, antifungal, anti-epileptic, and anti-inflammatory [71].

**Figure 20:**
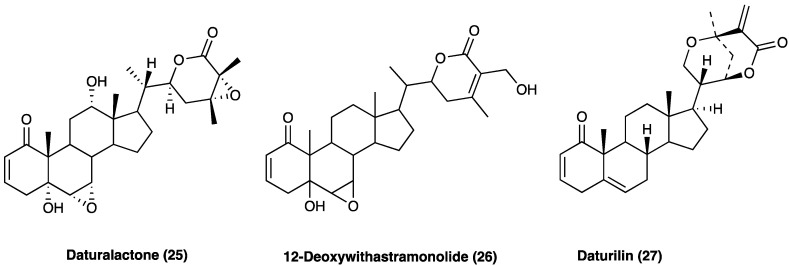
Daturalactone (D1), 12-deoxywithastramonolide (D23), and daturilin (D27) chemical structures.

A study conducted by Chandan et al. [72] identified three lactones, daturalactone (**25**, D1), 12-deoxywithastramonolide (**26**, D23), and daturilin (**27**, D27), as C28 steroids based on an ergostane skeleton (Figure 20) that showed inhibition of NO and pro-inflammatory cytokines (IL-1, IL-6, and TNF) released by LPS-activated J774A.1 macrophage. D1, D23, and D27 (20 mg/kg) were able to reduce pain and inflammation in tail-flick, acetic acid-induced writhing, vascular permeability assays, and carrageenan-induced rat paw edema in mice. Docking analysis showed that these three compounds actively bind to COX-1, COX-2, LOX-1, NF-κB, and iNOS, validating their anti-inflammatory effects.

### 3.17. Glycyrrhizin, Carbenoxolone, Licochalcone A, Isoliquiritigenin, and Isoliquiritin

Licorice, also known as kanzoh, gan-cao, sweet root [92] and yasti-madhu, is one of the most popular traditional herbal medicines in the world [93]. There are approximately 29 species of *Glycyrrhiza* (Fabaceae) worldwide, including 15 species with medicinal value. These species occur on all continents, except Antarctica, across 41 countries. Only one licorice is recorded in Indian pharmacopeia, two species are recorded in the US and Japanese pharmacopoeias, and three species are recorded in most national pharmacopoeias: *Glycyrrhiza glabra* L., *Glycyrrhiza uralensis* Fisch. ex DC. and *Glycyrrhiza inflata* Batalin. These three medicinal licorice species are mainly distributed in Eurasia, especially Central Asia. The main morphological differences between them are in leaves, inflorescences, pods, and seeds, and they can be distinguished by ITS and psbA-trnH sequences [94]. *G. uralensis* Fisch. ex DC., in combination in equal amounts with white peony root (*Paeoniae lactiflora* Pall., Paeoniaceae), is a well-known Chinese herbal formula (Shaoyao-Gancao Decoction, Shakuyaku-Kanzo-to in Japanese), commonly used to relieve myalgia, arthralgia and neuropathic pain, including paclitaxel [95], and CCI neuropathy [96].

The roots and rhizomes of licorice have long been used worldwide as a natural sweetener and traditional medicine used mainly for the treatment of peptic ulcer, hepatitis C, and pulmonary and skin diseases, although clinical and experimental studies suggest that it has several other useful pharmacological properties such as anti-inflammatory, antiviral, antimicrobial, antioxidative, anticancer, immunomodulatory, hepatoprotective, and cardioprotective [97]. Conventional extraction methods for licorice include ultrasonication, heat reflux, dispersive liquid–liquid micro-extraction, and molecularly imprinted solid-phase extraction. *G. glabra* L. has the highest concentration of triterpenoids and *G. uralensis* Fisch. ex DC. has the highest concentration of flavonoids [98]. Licorice constituents such as glycyrrhizin (**28**, GLA, Figure 20) and its derivatives (**29–33**), and other licorice-derived compounds, such as glabridin and isoliquiritigenin exert these effects via a range of mechanisms, including HMGB1 inhibition, gap junction blockade, and α2_A_-adrenoceptor antagonist.

#### 3.17.1. Glycyrrhizin

**Figure 21:**
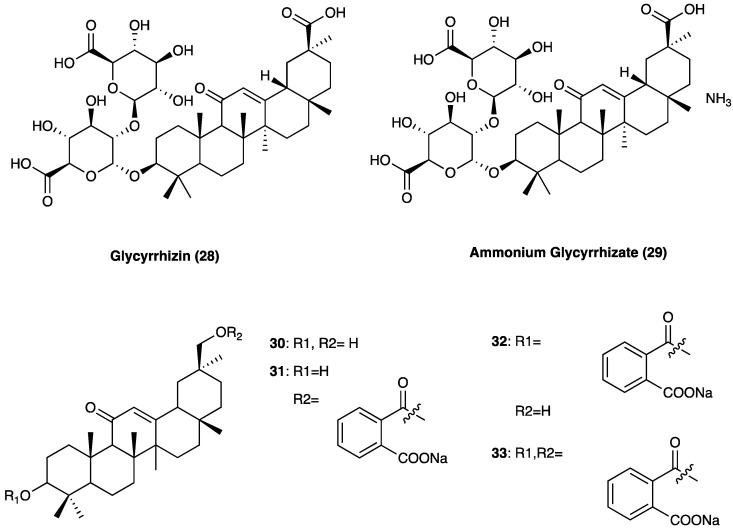
Structure of glycyrrhizinic acid and its derivatives.

Glycyrrhizin (**27**), also known as glycyrrhizic acid (Figure 21), is a triterpenoid saponin and is the main active compound.

It constitutes approximately 10% of the licorice root dry weight, where it is present as a mixture of potassium, calcium, and magnesium salts. Glycyrrhizin is a β-amyrin-type triterpenoid saponin, which numerous preclinical and cell studies report to have antiviral, neuroprotective, and potent anti-inflammatory properties. In Japan, glycyrrhizin has been used for more than 60 years to treat human chronic hepatitis. In STZ-induced neuropathic pain, glycyrrhizin treatment (five days/week for four weeks at a dose of 50 mg/kg per day i.p.) inhibited the increases in TLR4, NLRP3, and CXCR4 expressions and improved mechanical and thermal pain threshold. Immunohistochemical studies revealed that glycyrrhizin prevented the release of HMGB1 as well as H3K9 acetylation [99].

Several glycyrrhizin derivatives have also been studied in neuropathic pain models. In particular, ammonium glycyrrhizinate (**28**) was proven to have a cytoprotective effect on the neuroblastoma SH-SY5Y cell line after high-glucose administration. Also, in an in vivo experiment, a short-repeated treatment with ammonium glycyrrhizinate (i.p. injected at the dose of 50 mg/kg, 15, 17, and 19 days after the STZ administration) was able to attenuate neuropathic hyperalgesia in STZ-induced diabetic mice [100]. In a study by Akasaka et al. [101] several synthetic derivatives (Figure 20) of glycyrrhizin were evaluated and it was observed that the disodium salt of olean-11,13(18)-dien-3β,30-*O*-dihemiphthalate (**32**) inhibited the mobilization of Ca^2+^ induced by substance P, neurokinin A, and neurokinin B in CHO-K1 cells expressing the human NK1, NK2, and NK3 receptors, respectively. In the capsaicin inflammatory pain model, compound **32** suppressed flinching behavior in a dose-dependent manner, and it was also effective in suppressing pain-related behaviors in the late phase of the formalin test and reducing thermal hyperalgesia in the neuropathic pain state caused by sciatic nerve injury.

#### 3.17.2. Carbenoxolone

**Figure 22:**
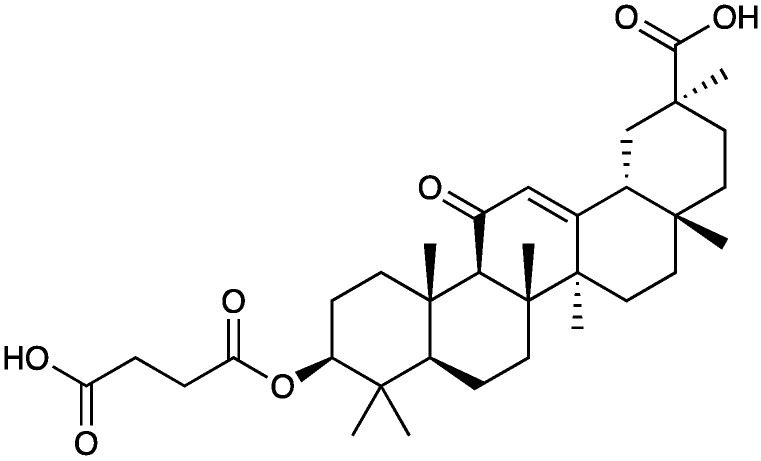
Chemical structure of carbenoxolone.

Carbenoxolone (**34**, Figure 22) is a glycyrrhetinic acid synthetic derivative with a steroid-like structure (Figure 21) used for the treatment of peptic, esophageal, and oral ulceration and inflammation. It exerts its analgesic effect by decoupling the gap junction.

When i.t. administered (1, 5, and 25 μg) in SCI neuropathic pain mice during the induction period, performed twice a day at 8 A.M. and 6 P.M. on postoperative days 0 to 5, carbenoxolone dose-dependently attenuated the development of bilateral thermal hyperalgesia and mechanical allodynia and significantly reduced bilateral increase in GFAP-immunoreactive staining and the number of pNR1-ir cell profiles in spinal cord dorsal horn. In contrast, carbenoxolone treatment during the maintenance phase on postoperative days 15 to 20 had no effect on the established thermal hyperalgesia and mechanical allodynia, nor on spinal GFAP expression or the number of pNR1-ir cell profiles [102]. Anyway, repetitive i.t. carbenoxolone treatment through days 14–16 post-SNL did not cause acute desensitization or tachyphylaxis to its pain-inhibiting effect [103]. Carbenoxolone has also been demonstrated to attenuate facial mechanical hypersensitivity as well as the accompanying central sensitization of functionally identified malate dehydrogenase nociceptive neurons induced by trigeminal nerve injury [104].

#### 3.17.3. Licochalcone A

**Figure 23:**
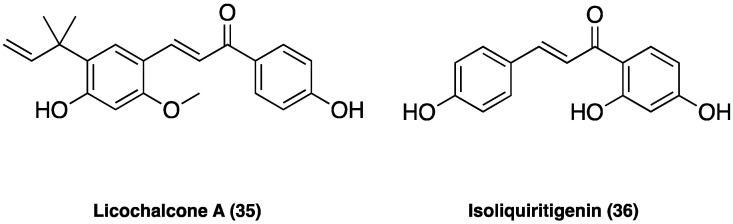
Chemical structure of licochalcone A and isoliquiritigenin.

Licochalcone A (**35**, Figure 23), the main phenolic constituent of licorice, has a variety of bioactivities, such as anti-inflammatory, neuroprotective, and anti-cancer properties. It decreased the expression of inflammatory factors by inhibiting MAPK and the AKT/NF-kB pathways and repaired the blood–milk barrier damage caused by inflammation. Licochalcone A (**35**) also inhibited JNK1, providing neuronal protection against excitotoxic insults [105].

In CCI neuropathic pain, i.p. administered (after CCI surgery from the 4-th day to the 10-th day twice daily at the dose of 1.25, 2.50, and 5.00 mg/kg), it significantly attenuated pain in a dose-dependent manner, ameliorating the mechanical withdrawal thresholds and thermal withdrawal latencies. Additionally, licochalcone A (**35**) administration also effectively blocked microglia activation, suppressed p38 phosphorylation, and the release of inflammatory factors such as TNF, Il-1β, and IL-6 [106].

#### 3.17.4. Isoliquiritigenin

Isoliquiritigenin (**36**, Figure 23) is a flavonoid from *G. glabra* L., known to have various pharmacological activities, including antibacterial and anti-inflammatory effects. Isoliquiritigenin (**36**) has been reported to inhibit Na_v_1.4, Ca^2+^ channels, and NMDA receptors, thus, presumably supporting its analgesic effect. In small- and medium-sized cultured trigeminal ganglion neurons, the compound suppressed, dose-dependently, Na_v_ currents in many neurons (78%) and K_v_ currents in all neurons. In behavioral experiments, local treatment with isoliquiritigenin (**36**) suppressed nociceptive behaviors in response to oral ulcer development or to nociceptive TRP channel agonists [107]. Additionally, in vivo isoliquiritigenin (**36**) could cause a significant reduction in the acetic acid-induced writhing response and hot-plate test [108].

#### 3.17.5. Isoliquiritin

**Figure 24:**
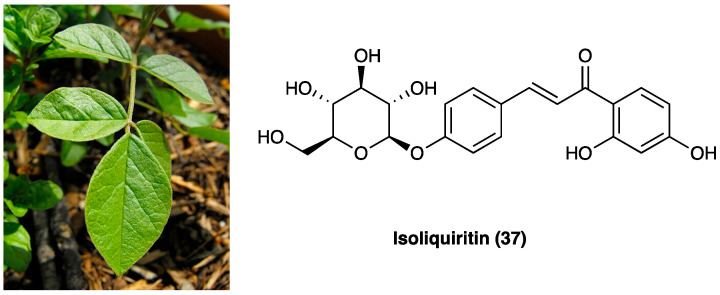
*Glycyrrhiza uralensis* Fisch. ex DC. and chemical structure of isoliquiritin.

Isoliquiritin (**37**, Figure 24) is a flavonoid glycoside compound from *G. uralensis* Fisch. ex DC. (Figure 24) that possesses a variety of biological and pharmacological effects, such as anti-tumor, pro-angiogenic, antifungal, antigenotoxic, and neuroprotective actions, suggesting disease-modifying and health-promoting properties. Additionally, it possesses antidepressant-like properties [109].

In CCI neuropathic pain, chronic treatment with isoliquiritin (**37**) (5, 15, and 45 mg/kg, p.o., twice per day for two weeks) ameliorated, dose-dependently, behavioral hyperalgesia to thermal (heat) stimuli and allodynia to tactile stimuli. Isoliquiritin (**37**) antihyperalgesic and antiallodynic actions were totally abolished by the chemical depletion of spinal serotonin by *p*-chlorophenylalanine but potentiated by 5-HTP (a precursor of 5-HT). Consistently, isoliquiritin-treated neuropathic mice showed escalated levels of spinal monoamines, especially 5-HT, with depressed MAO activity. Moreover, isoliquiritin antihyperalgesia and antiallodynia were preferentially counteracted by systematically or spinally 5-HT1A receptor antagonist WAY-100635. Isoliquiritin (**37**) was also able to correct co-morbid behavioral symptoms of depression and anxiety evoked by neuropathic pain [110].

### 3.18. Agarwood

**Figure 25:**
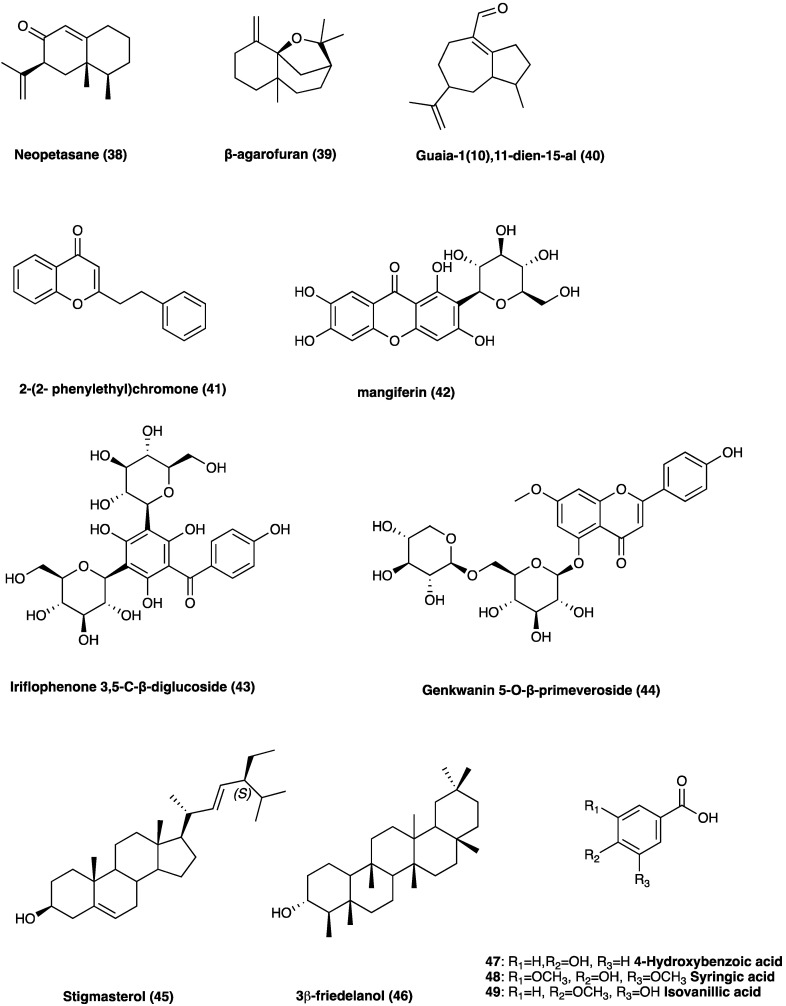
Chemical structures of major compounds found in agarwood.

Agarwood is the wood of different species of the genus *Aquilaria* Lam. (Thymelaeaceae), especially splint wood containing penetrated black resin. It has been employed in traditional medicines as an aphrodisiac, sedative, cardiotonic, and carminative [18]. The economic interest in agarwood has been directed towards its heavy and dense resin, formed in the tissues of the stem after an injury. Recently, several pharmacological actions of agarwood have been investigated, such as anti-diabetic, anti-cancer, cytotoxic, antioxidant, and especially anti-inflammatory [19,20,21,22], demonstrated by pre-clinical studies. In Figure 25, the chemical structures of the major compounds found in agarwood are presented: neopetasane (eremophilane) (**38**), β-agarofuran (**39**), (−)-guaia-1(10),11-dien-15-al (**40**), 2-(2-phenylethyl)chromone (**41**), mangiferin (**42**), iriflophenone 3,5-*C*-β-diglucoside (**43**), genkwanin 5-*O*-β-primeveroside (**44**), stigmasterol (**45**), 3β-friedelanol (**46**), 4-hydroxybenzoic acid (**47**), syringic acid (**48**), and isovanillic acid (**49**) [19].

The latest investigations on agarwood have disclosed that sesquiterpenes and 2-(2-phenylethyl) chromone derivatives are the two predominant constituents [22]. In particular, the 2-(2-phenylethyl)chromone, with a phenylethyl substituent at the C-2 position, is involved in its anti-inflammatory activity [24]. The ethylacetate soluble fraction from a 95% EtOH extract of the resinous wood of *Aquilaria sinensis* (Lour.) Spreng. was found to inhibit NO production in LPS-stimulated RAW264.7 cells. Furthermore, nine undescribed sesquiterpene derivatives were isolated and identified from *Aquilaria malaccensis* Lam. All nine compounds were screened for their anti-inflammatory activities, and one of these showed potential NO inhibitory effects [25,26].

### 3.19. Leucodin and α-Santonin

**Figure 26:**
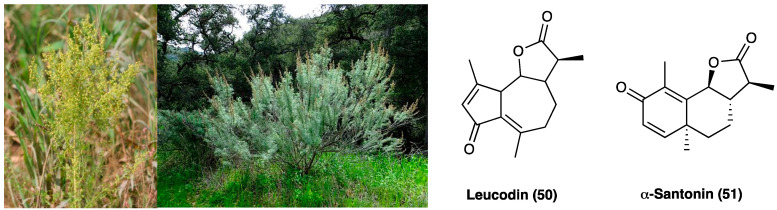
*Artemisia annua* L., *Artemisia californica* Less. and chemical structures of leucodin and α-santonin.

Plants of the Asteraceae family displayed mainly antimicrobial and analgesic activities, and for this reason, they were used in traditional medicine for long time. The most known is *Artemisia annua* L. (Asteraceae), used for its anti-malarian activity due to a sesquiterpene lactone, named artemisinin [38]. *Artemisia californica* Less. (Asteraceae) (Figure 26) was an important remedy for headache. *A. californica* Less.’ leaves and stems decoction were used externally for colds, asthma, and arthritis [39]. In recent years, an alcoholic liniment of this plant showed encouraging data in pain patients affected by arthritis, muscle and ligament strains, bruises, broken bones, low back pain, and cancer. This plant is rich in flavonoids, terpenoids, alkaloids, phenols, and polyacetylenes. These constituents can inhibit NO production, opening the possibility of anti-inflammatory uses [40]. Among the sesquiterpene lactones, leucodin (**50**, Figure 26) presents a cyclopentadienone rigid ring system that may react with thiols in proteins to provide its biological activity. Literature studies report that leucodin (**50**) can inhibit COX-2 and inducible NO synthase, thus showing an anti-inflammatory profile. Another constituent of *Artemisia* species is a molecule called α-santonin (**51**, Figure 26), used as an anthelminthic, then removed from the market for its hazards to patients’ health.

To date, α-santonin (**51**) is a promising agent for the synthesis of new derivatives with anti-inflammatory and cytotoxic activity. It showed, in fact, strong anti-inflammatory, antipyretic and analgesic properties against carrageenan-induced edema in rat paw. Nowadays, the specific mechanism of action is not entirely clear, but it may be due to the suppression of kinin and prostaglandin formation [41].

### 3.20. β-Caryophyllene

**Figure 27:**
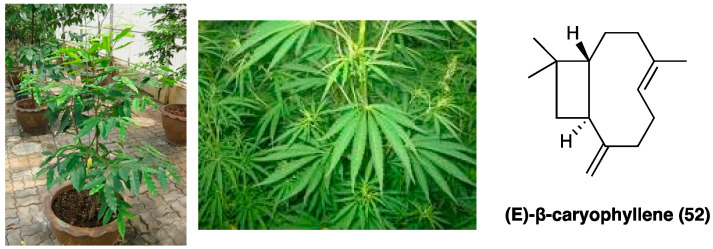
*Aquilaria crassna* Pierre, *Cannabis sativa* L. and (*E*)-β-caryophyllene structure.

The sesquiterpene hydrocarbon (*E*)-β-caryophyllene (**52**, Figure 27) is one of the most interesting natural compounds employed for inflammatory and neuropathic pain treatment. Regarding the chemical structure, (*E*)-β-caryophyllene (**52**) is a bicyclic sesquiterpene found in the essential oils of many different spice and food plants such as *Aquilaria crassna* Pierre (Thymelaeaceae) and *Cannabis sativa* L. (Cannabaceae). Moreover, (*E*)-β-caryophyllene is known as the major component of marijuana essential oil. In particular, it is a secondary metabolite belonging to the group of terpenes present in both vegetative and reproductive parts. It is mainly involved in plant defense and attraction. It is approved to be used as a natural flavoring agent by the FDA.

Recently, much evidence has suggested that it has a protective role, also with beneficial effects against different diseases, especially in pathologies characterized by chronic inflammation [29]. Specifically, (*E*)-β-caryophyllene (**52**) is a CBR2 receptor selective full agonist. Several studies showed that (*E*)-β-caryophyllene (**52**) is able to reduce the expression of cytokines and attenuate mechanical allodynia in animal models of paclitaxel- and diabetic-induced neuropathic pain. (*E*)-β-caryophyllene (**52**) suppressed cytokine expression, phospho-ERK1/2 levels, and decreased COX-2 and iNOS expression, which could suppress NF-κB activation and consequently promote analgesia [30]. In a mouse model of antiretroviral drug-induced neuropathic pain (*E*)-β-caryophyllene (administered by oral gavage at a loading dose of 50 mg/kg and a maintenance dose of 25 mg/kg twice daily for 5 days or at a dose of 25 mg/kg once after neuropathic pain) decreased mechanical allodynia [31], and in CCI-induced neuropathic pain, dose- and time-dependently, in both male and female mice, it showed a good potency in reversal thermal hyperalgesia in comparison with mechanical allodynia [32]. In the formalin test, (*E*)-β-caryophyllene (**52**), orally administered at the dose of 5 mg kg^−1^, is antinociceptive in the late phase of the formalin test after acute treatment [33] without gastric damage. The data related to both in in vivo and in vitro studies show that (*E*)-β-caryophyllene (**52**) is a good candidate in the treatment of chronic inflammation of its specific molecular targets and low toxicity. Mainly, specific (*E*)-β-caryophyllene-mediated CBR2 receptor activation plays an anti-inflammatory activity through the modulation of NF-κB and PPARγ [34]. (*E*)-β-caryophyllene is also characterized by good lipophilicity, so it easily penetrates cell membranes [35]. Moreover, literature data report that the inclusion complex containing (*E*)-β-caryophyllene (**52**) and β-cyclodextrin significantly increased the oral bioavailability of the drug over the free (*E*)-β-caryophyllene.

Recently, (*E*)-β-caryophyllene (**52**) was combined with other natural products, such as carnosic acid, in a single formulation named Noxiall^®^ used as a food supplement. Carnosic acid, present in *Rosmarinus officinalis* L. (Lamiaceae) and *Salvia officinalis* L. (Lamiaceae), has antioxidant and anti-inflammatory properties. Its anti-inflammatory action is based on COX-2 inhibition, IL-1β and TNFα reduction, and leukocyte infiltration attenuation into the damaged tissues. The potential analgesic and anti-inflammatory efficacy of this mixture was evaluated in animal models of neuropathic pain. The results showed that this formulation is able to reduce the mechanically-induced allodynia in CCI mice. This effect is dose-dependent, is maintained for the full treatment period and not subjected to rapid tolerance development. The formulation efficacy was comparable to that of gabapentin and pregabalin, approved as first-line treatment of neuropathic pain. Moreover, if co-administered with pregabalin, it enhanced the efficacy of the gabapentinoid, showing a potential role in the treatment of neuropathic pain [36]. A probable synergistic effect of the combination of (*E*)-β-caryophyllene (**52**) and docosahexaenoic acid on the modulation of inflammatory pain responses was also evaluated. The obtained data showed the combination of the two compounds caused a marked reduction of formalin-induced pain responses [37].

### 3.21. Crocin

**Figure 28:**
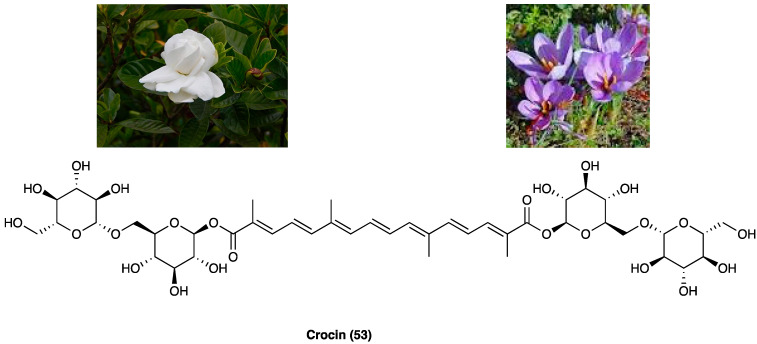
*Gardenia jasminoides* J. Ellis, *Crocus sativus* L. and the structure of crocin.

Crocin (**53**, Figure 28) is the digentiobiosyl ester of all-*trans*-crocetin (8,8′-di-apocarotene-8,8′-dioic acid) ester that is the major yellow pigment in gardenia yellow and saffron, isolated by *Gardenia jasminoides* J. Ellis (Rubiaceae) fruits and *Crocus sativus* L. (Iridaceae) stigmas, respectively [75]. Concerning the chemical structure, crocin is a diester composed of the disaccharide gentiobiose and the dicarboxylic acid crocetin. These constituents are radical scavengers [76].

A range of scientific evidence shows that crocin (**53**) possesses various pharmacological effects, such as protection against cardiovascular diseases, tumoricidal action, neuroprotective, antioxidant as well as anti-inflammatory properties [77,78]. Specifically, its antioxidant properties are mediated through the modulation of GPx, GST, CAT, and SOD. The literature shows that crocin has a relevant anti-inflammatory activity in several human systems, such as respiratory, nervous, gastrointestinal, cardiovascular, urogenital and musculoskeletal [79]. Experimental evidence suggests that antioxidant/anti-inflammatory properties of crocin contribute to its anti-nociceptive effects. Crocin (**53**) possesses beneficial effects against STZ-induced cold allodynia and edema in rats. Also, in rats, intra-fourth ventricle injection of crocin (10 and 40 µg/rat) in a model of capsaicin-induced orofacial pain, as well as i.p. injections (12.5 and 25 mg/kg) in i.pl. injection in formalin-induced pain, displayed anti-nociceptive effects. Crocin (**53**) also enhanced morphine-induced antinociception [80]. A recent study also reported that crocin (**53**) showed beneficial effects during critical phases of chronic pain development, probably reestablishing sensory and motor neurons in the rats with SCI, without effect on spinothalamic neurons [81]. The administration of a CB receptor antagonist reduced the antinociceptive effects of crocin, suggesting a CB receptor involvement [82]. In another study, it was shown that crocin reduced CCI neuropathic pain, but was reversed by atropine, demonstrating the interaction with the cholinergic system. Additionally, different studies have highlighted that simultaneous administration of crocin (**53**) and morphine after induction of CCI could attenuate morphine tolerance, suggesting a good strategy to prevent the development of morphine tolerance in neuropathic pain treatment [83].

### 3.22. Kirenol

**Figure 29:**
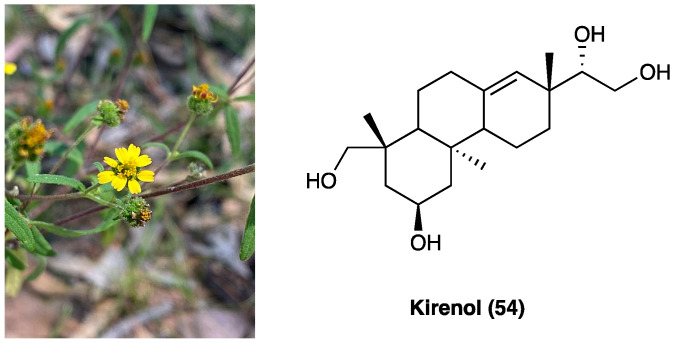
*Siegesbeckia orientalis* L. and kirenol structure.

*Siegesbeckia orientalis* L. (Asteraceae) is an annual herb mainly distributed in Central China. Its aerial part has been usually employed in the treatment of arthritis, hypertension, malaria, neurasthenia, and snakebite. Traditional Chinese medicine reports that *S. orientalis* L. is topically used as an analgesic and anti-inflammatory agent in the treatment of snake bites, cutaneous disorders, and rheumatic arthritis. The aerial part of this plant is mainly composed of ent-kaurane, ent-pimarane type diterpenoids, and sesquiterpenoids. Kirenol (**53**, Figure 29), the main ent-pimarane type diterpenoid present in *S. orientalis* L., is defined as the most important anti-inflammatory and anti-rheumatism active constituent. It was selected as the reference substance to quantify and qualify *S. orientalis* L. in the Chinese Pharmacopoeia. In recent years, the topical effects of kirenol (**54**), have been investigated to evaluate its inflammatory and analgesic response in vivo, employing the carrageenan-induced rat paw edema model and formalin test.

Data demonstrated that kirenol (**54**) presents analgesic and anti-inflammatory activities and can be employed as a remedy for topical pain and inflammation [175]. Recently, a new study was carried out to clarify the mechanisms underlying the anti-inflammatory and analgesic influence of active components of *Siegesbeckia pubescens* Makino (Asteraceae). This study demonstrated that its monomer active components (kirenol, darutoside, and hesperidin) can inhibit inflammatory infiltrates and COX-2 expression nociceptive stimulus-induced effects, thus, exhibiting significant anti-inflammatory and analgesic effects [176].

### 3.23. Geniposide

**Figure 30:**
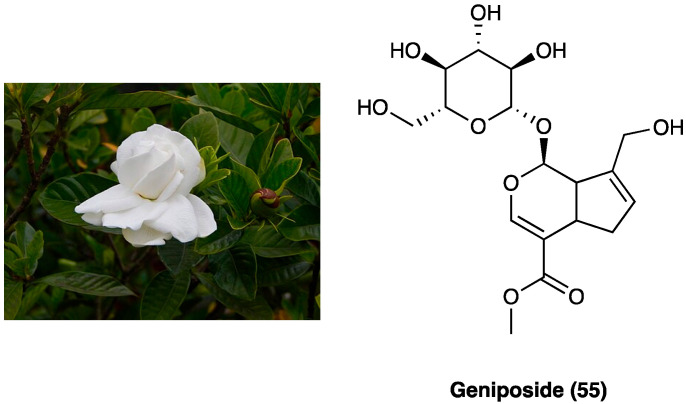
*Gardenia jasminoides* J.Ellis and geniposide structure.

Geniposide (**55**, Figure 30), a iridoid (monoterpenoid) glycoside, is one of the main glycosides of *Gardenia jasminoides* J.Ellis (Rubiaceae) (Figure 30), which is mainly employed in Chinese traditional medicine for its homeostatic, antiphlogistic, antinociceptive, and antipyretic properties [84].

Recently, it was demonstrated that the activation of spinal GLP-1Rs, specifically expressed in spinal dorsal horn microglial cells and up-regulated after peripheral nerve injury [85] with the peptidic agonist exenatide produced antinociception in chronic pain. Spinal GLP-1Rs could be a potential target for chronic pain treatment. In a recent study, the antinociceptive activities of geniposide (**55**), as a possible small molecule with GLP-1R agonist profile, was evaluated. Geniposide (**55**) is able to exert complete protection against hydrogen peroxide-induced oxidative damage in humans, showing a protective action against oxidative stress. S.c. (3, 10, 30, 100, or 300 mg/kg) and p.o. geniposide (**55**) (10, 30, 100, 300, or 1000 mg/kg 1 h before formalin) dose-dependently blocked the formalin-induced tonic but not the acute flinching response. It was suggested that geniposide (**54**) is able to produce antinociception during persistent pain by activating the spinal GLP-1Rs and that the iridoids, represented by geniposide (**55**), are orthosteric agonists of GLP-1Rs [84]. Geniposide (**55**) also reduces the expression of inflammatory cytokines and could increase the pain threshold in CCI rats by inhibiting TNF-α expression in the ipsilateral or contralateral dorsal root ganglia. It was also found that geniposide (**55**) may exert anti-inflammatory effects through EGFR and that geniposide (**55**) reduced the expression of inflammatory factors and improved pain threshold in CCI by inhibiting Ca^2+^ channel activity [86].

### 3.24. Scopoletin and Spinasterol

**Figure 31:**
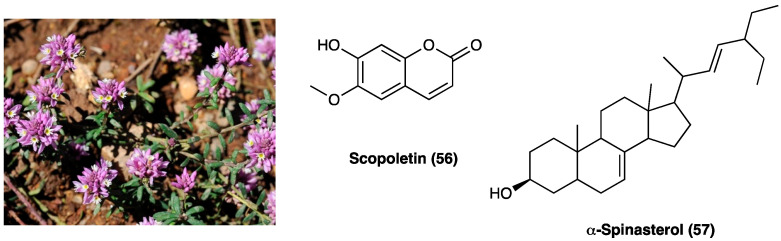
*Polygala sabulosa* A. W. Bennett and structures of major constituents in *Polygala*.

*Polygala sabulosa* A. W. Bennett (Polygalaceae) is a small herb diffused in the Southern Meridional Highlands of Brazil. The plants of the genus *Polygala* are employed for disorders of the bowel and kidney as a tonic remedy, topical anesthetic, and expectorant. Chemical studies carried out on different species of the genus *Polygala* showed the presence of different classes of constituents, such as coumarins, saponins, lignans, flavonoids, and mainly xanthones (Figure 31). The possible antinociceptive activity of i.p. injection of the hydroalcoholic (CH_2_Cl_2_, EtOAc, n-BuOH) extract (1–100 mg kg^−1^), aqueous fraction and pure compounds (0.001–10 mg kg^−1^) obtained from the whole plant *P. sabulosa* A. W. Bennett was investigated in acetic acid-induced visceral pain in mice, where they caused a relevant dose-dependent antinociceptive response.

The CH_2_Cl_2_, EtOAc, and n-BuOH fractions were more potent than the hydroalcoholic extract and aqueous fraction. Scopoletin (**56**) and spinasterol (**57,** Figure 31) have been identified as the active principles of the plant, indicating these constituents or their derivatives for possible use in the development of new analgesic drugs [131]. Moreover, it was shown that the antinociceptive effects of *P. sabulosa* A. W. Bennett hydroalcoholic extract are associated with glutamatergic transmission inhibition, which is pro-inflammatory cytokines-dependent [132].

### 3.25. Saikosaponin A

**Figure 32:**
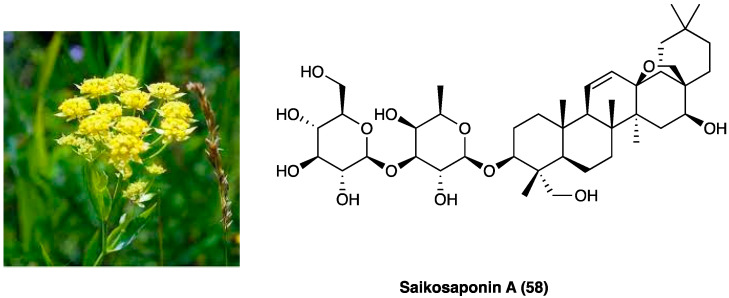
*Bupleurum chinense* DC. and structure of saikosaponin A.

Radix Bupleuri (Figure 32), named also with the Chinese name Chai hu, is the dried root of *Bupleurum chinense* DC., an herbal plant of the Apiaceae family, distributed mainly in Hubei and Sichuan Provinces of China. Traditionally, it has been employed for the treatment of the common cold with fever, hepatitis, kidney syndrome, and inflammatory diseases [44]. Saikosaponins are the major chemical constituents of Radix Bupleuri, and they possess anti-inflammatory, immune-regulating, antibacterial, and antiviral activities [45]. Among saikosaponins, saikosaponin A, a triterpenoid saponin (**58**, Figure 32) is the major active constituent with anti-inflammatory activity.

Zhou et al. evaluated the effects of saikosaponin A on CCI neuropathic pain induced [46] and the involved molecular mechanisms. The results showed that saikosaponin A (6.25, 12.50, and 25.00 mg/kg i.p., once daily for 14 days) is able to reverse CCI-decreased mechanical and thermal withdrawal threshold, to inhibit CCI-increased levels of TNF-α, IL-1β, and IL-2, and to decrease the expression of p-p38 MAPK and NF-kB, able to induce the expression of various pro-inflammatory genes, including those encoding cytokines and chemokines, in CCI-induced in the spinal cord.

### 3.26. (−)-Spectaline

**Figure 33:**
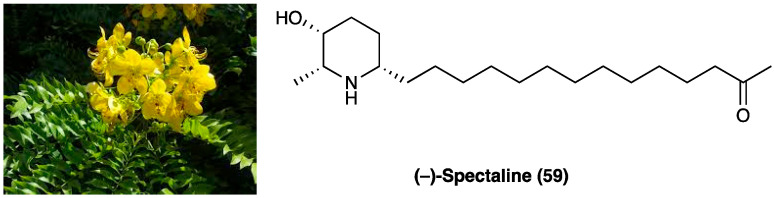
*Cassia leptophylla* Vogel and (−)-spectaline structure.

(−)-Spectaline (**59**, Figure 33) is a piperidine alkaloid isolated from *Cassia leptophylla* Vogel (Fabaceae) (Figure 33), mainly used in traditional medicine for antimicrobial, laxative, antiulcerogenic, analgesic, and anti-inflammatory properties [47].

The possible antinociceptive activity of (−)-spectaline (**59**) was investigated on capsaicin-induced pain. Hot plate and tail flick tests were also performed to determine whether the antinociceptive effect of (−)-spectaline (**59**) was due to a central or peripheral mechanism. The obtained results showed that (−)-spectaline (**59**) did not induce a pain latency increase in the hot plate and tail flick test. Conversely, it was observed that (−)-spectaline (**59**) (1.6 mg/paw) affected capsaicin-induced pain, suggesting that this compound may directly interact with the primary afferent-mediated transmission of pain signals and that it could directly interact with the vanilloid system or with excitatory amino acids by binding to their receptors [48].

### 3.27. Fisetin

**Figure 34:**
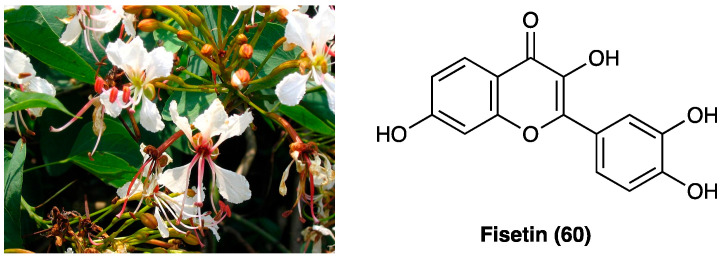
*Bauhinia glauca* ssp. *hupehana* (Craib) T.C.Chen and chemical structure of fisetin.

*Bauhinia glauca* (Figure 34) belongs to the Leguminosae family, consisting of about 300 species, whose stem bark and leaves are widely used in oriental medicine to treat different inflammatory disorders like backache, rheumatic arthritis, and hemostasis swelling. *B. glauca* ssp. *hupehana* (Craib) T.C.Chen was reported, for the first time by Xu J. et al., for the pain-relief action both in chemical and thermal nociceptive stimuli in mice [42]. They used an ethanol extract of aerial parts (from which were isolated different chemical compounds: peperomin B and fisetin (**60**), followed by quercetin (**66**), luteolin, and garbanzol) that, at a dose of 100 mg/kg, p.o., administered in acetic acid-induced writhing and hot-plate test in mice, exhibited an anti-nociceptive effect. Interestingly, it was also found involvement of ATP-sensitive K^+^ channel pathway, since only glibenclamide, but not naltrexone pretreatment, was able to revert its antinociceptive activity, thus, excluding opioid system involvement. Among the active principles of *B. glauca* ssp. *hupehana* (Craib) T.C.Chen, fisetin (3,3′,4′,7-tetrahydroxyflavone) (**60**, Figure 34), a flavonoid compound able to overcome the BBB, is one of the most studied for its analgesic, antidepressant, and neuroprotective action.

It was deeply investigated fisetin (**60**) analgesic action in the CCI model (15 and 45 mg/kg administered p.o. twice a day for 3 weeks) [43]. They demonstrated a significant reduction of thermal, but not mechanical hyperalgesia and discovered that the compound interacts with the serotonin system, inhibits MAO-A activity, and activates 5-HT_7_ receptors at the spinal but not at the supraspinal level. They also confirmed fisetin (**60**) antihyperalgesic action (15–45 mg/kg) on thermal hyperalgesia and mechanical allodynia in diabetic neuropathic pain model, by its antioxidant activity in dorsal root ganglia and spinal cord of mice, but not supraspinal GABA_A_ receptor involvement.

### 3.28. Betulinic Acid

**Figure 35:**
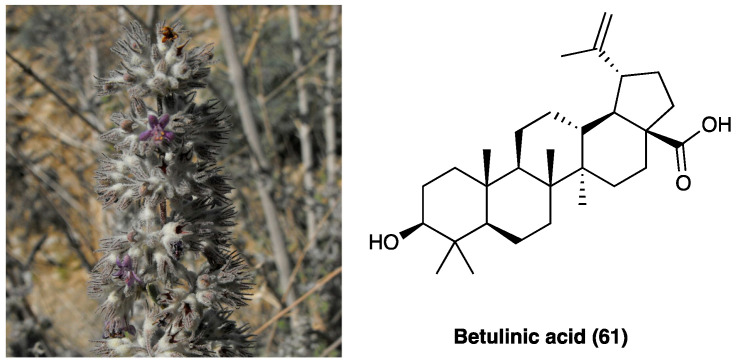
*Hyptis emoryi* Torr. and betulinic acid structure.

*Hyptis emoryi* Torr., belonging to the Lamiaceae family, is a perennial shrub widely present in many U.S. states such as Arizona and Colorado, also known as desert lavender. It is used as an anesthetic to treat gastric disorders. Essential oil is rich in flavonoids and lignans, while the major chemical compound of the aerial part is betulinic acid (**61**, Figure 35), a pentacyclic triterpenoid with anti-bacterial, anti-cancer, anti-HIV, anti-diabetic, and anti-inflammatory activities, but also with protective action against Alzheimer’s disease and atherosclerosis.

It was studied for its analgesic effect due to interaction with Ca^2+^ channels, especially N- and T-type. Bellampali et al. found that only betulinic acid (**61**) was obtained from a *H.s emoryi* Torr. extract in comparison with oleanolic and ursolic acid, was able to inhibit evoked Ca^2+^ influx [111]. They demonstrated that betulinic acid (**61**) principally interacts with Cav3.2 (T-type) and Cav2.2 (N-type), which activity is increased in afferent pain fibers in several chronic pain conditions, also resulting in a decreased depolarization-evoked CGRP release. It does not interact either with the opioid receptor, as demonstrated by binding studies, nor with the Na^+^ voltage channel. In a different animal model, such as chemotherapy- and HIV-induced neuropathy, i.t. injection of 2 μg/5 μL of betulinic acid significantly reverted mechanical allodynia compared to the control group, with a peak at about 120 min after its administration in both assays. Furthermore, in PSNL, a model of neuropathy that involves Cav2.2 channel’s plasticity, i.t. injection of 2 μg/5 μL of betulinic acid (**61**), after 7 days PSNL induction, significantly reverted mechanical allodynia 2 and 3 h after administration, in comparison with the vehicle group. Thomas et al. confirmed the anti-inflammatory activity of betulinic acid (**61**), isolated from *Uapaca staudtii* Pax (Phyllanthaceae) ethanol extract, using fresh egg albumin-induced hind-paw and xylene-induced ear edema in mice [112]. However, betulinic acid (**61**) shows poor water solubility and a short half-life, hence, it is not possible to use a systemic administration route.

### 3.29. Quercetin and Kaempferol

*Albizia anthelmintica* Brongn is a tree widely diffused in tropical Africa and South America regions that belongs to the Fabaceae family, traditionally used to treat depression, cough, diarrhea, and rheumatism. *Albizia* species are rich in phenolic and triterpenoids saponins, known for their anti-inflammatory and antioxidant action.

**Figure 36:**
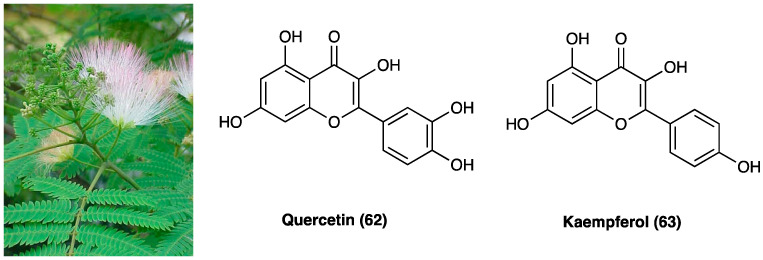
*Albizia anthelmintica* Brongn, quercetin and kaempferol structure.

Mohamed et al. isolated several compounds from *A. anthelmintica* Brongn alcoholic extract, such as gallic acid (**22**), quercetin (**62**), kaempferol (**63**), and their glucoside derivates (Figure 36). The ethanolic extract evaluation showed a dose-dependent antinociceptive effect in acetic acid-induced writhing test and an anti-inflammatory activity in carrageenan-induced hind paw edema. Isolated chemical compounds, quercetin (**62**) and kaempferol glucopyranoside exhibited a better scavenging activity compared to extract in diphenyl-picrylhydrazine assay [27].

Experiments performed on methanol leaf extract of *A. anthelmintica* Brongn focused on flavonoids and galloyl glucoside derivates, such as quercetin O-galloyl-glucoside that showed the best binding to several key inflammation enzymes like 5-LOX, COX-1 and COX-2 while, among phenolic compounds, eucomic acid exhibited the best binding value, but lower than the former compound. Moreover, they found that only the highest dose of the extract (400 mg/kg), orally administered, was effective in later phases of edema (up to 24 h), suggesting an effect on leukocyte migration. The lower dose (200 mg/kg) showed a significant antinociceptive effect in acetic acid-induced writhing in mice and a prolonged but delayed, effect in the hot-plate test. Finally, they demonstrated a similar antipyretic action for both doses in Brewer’s yeast induced pyrexia in mice up to 24 h after administration [28].

### 3.30. Incarvillateine

**Figure 37:**
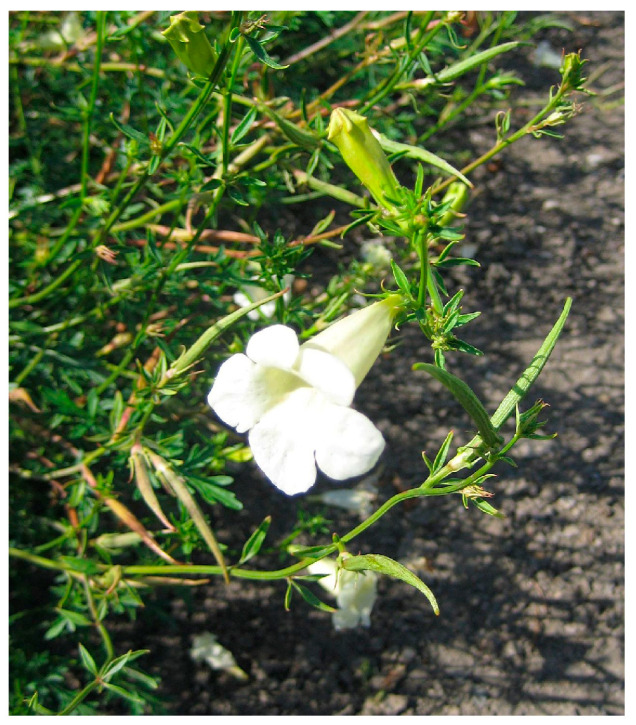
*Incarvillea sinensis* Lam.

*Incarvillea sinensis* Lam. (Bignoniaceae) (Figure 37), also known as Jiaohao, is a wild plant diffused in the northern area of China, conventionally used as crude drug in rheumatism management and relieving pain. This plant is characterized by several antinociceptive active principles.

**Figure 38:**
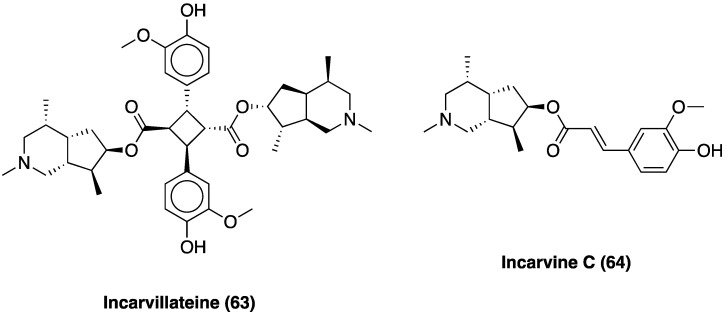
Structures of incarvillateine and incarvine C.

Over the years, several novel monoterpene alkaloids and macrocyclic spermine alkaloids have been discovered and characterized. One of these, a monoterpene alkaloid named incarvillateine (**63**, Figure 38) [113], was demonstrated to have a relevant antinociceptive effect in the formalin test. Incarvillateine (**63**) is obtained via dimerization of incarvine C (**64**, Figure 37), a hydroxycinnamate derivative of incarvilline. Its structure is characterized by a bicyclic piperidine moiety bearing five contiguous stereocenters [114].

The correlation between the incarvillateine structure (**63**) and antinociceptive activity was evaluated. The data clearly showed that an important factor in the antinociceptive effects was the presence of a monoterpene alkaloid and a dimeric structure carrying a cyclobutane ring in its component [115].

Incarvillateina (**63**) analgesic action was focused and incarvillateine, i.p. administered, exhibited a significantly dose-dependent antinociceptive effect in acetic acid-induced writing, confirming previous studies [116]. In SNI- and paclitaxel-induced neuropathic pain, incarvillateine (**63**, 20 mg/kg i.p.) showed a potent anti-allodynic effect without tolerance development. Moreover, they showed an anti-hyperalgesic and a mild but significant antiedema action in the CFA-induced paw model. The lack of effects in the hot-plate test, the lack of tolerance, and the lack of naloxone antagonism on incarvillateine (**63**) antinociceptive effects indicate no involvement of the opioid system in its analgesic action. Instead, the adenosine system, in particular the A_1_ receptor seems to be involved in its analgesic effect, as also confirmed by DMPX (3,7-dimethyl-1-propargylxanthine) administration [117].

Recently, it was highlighted that the derivatives of cinnamic acid dimers, which are structurally similar to incarvillateine (**63**), showed potent antinociceptive action. Therefore, incarvillateine analogs were synthesized using the cavitand-mediated photodimerization method, which utilizes a macromolecule (γ-cyclodextrin) to control the excited state reactivity of photoactive compounds. The pain response was evaluated by using formalin-induced licking behavior in the hind paws of mice. The results suggest that incarvillateine showed potent non-opioid antinociceptive action mediated predominantly through adenosine 3 receptor action [118]. On the other hand, Kim et al. also confirmed that incarvillateine monoeseter derivate showed a potent analgesic action related to the adenosine system, but their results indicated that only incarvillateine (**63**) suppressed locomotion, suggesting an A_2_ receptor involvement, also confirmed by DMPX antagonism [117].

**Figure 39:**
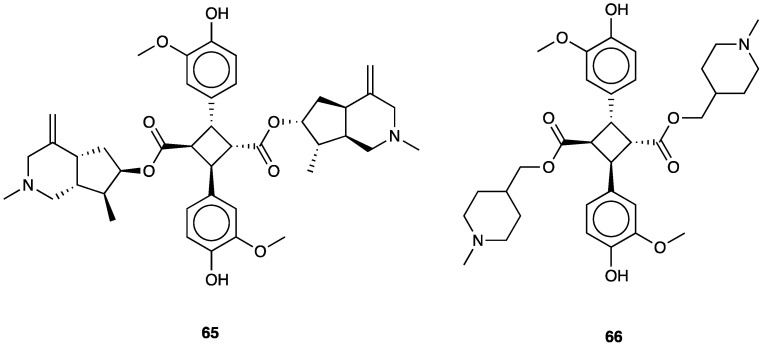
Structures of incarvillateine analogs.

Only in 2009 was it possible to totally develop an enantioselective synthesis of incarvillateine [119], and immediately, this compound attracted considerable attention. Seo et al. described an enantioselective synthesis of 7-epi-incarvillateine as the key intermediate to obtain (−)-incarvillateine and other components extracted by *I. sinensis* Lam., like (−)-incarvilline and (+)-incarvine C (stereoselective synthesis of 7-epi-incarvilline), although Wang et al. indicated that the cyclobutane ring is indispensable for the antinociceptive effect. Moreover, Huang et al. synthesized six analogs of incarvillateine (**63**) focusing on these two derivates **65** and **66** (Figure 39) that demonstrated a higher inhibitory activity than incarvillateine (**63**) in an acetic acid-induced test. Then, they evaluated both compounds in inflammatory and neuropathic pain models.

Results obtained in the formalin test showed that incarvillateine (**63**) showed a higher antinociceptive effect in both phases than two derivates, but in the SNI model, the two derivatives, especially compound **65**, showed a more powerful and longer-lasting anti-allodynic effect [120].

### 3.31. Quercetin

**Figure 40:**
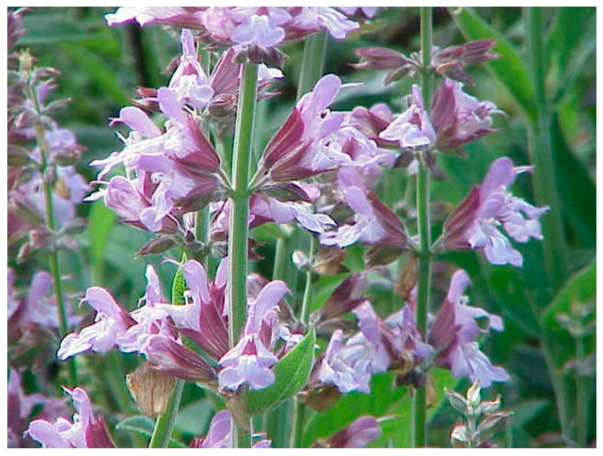
*Salvia officinalis* L.

Quercetin (**62**) 2-(3,4-dihydroxyphenyl)-3,5,7-trihydroxy-4H-chromen-4-one, present in *Salvia officinalis* L. (Lamiaceae) (Figure 40) is an element of flavonoid family compounds, which includes over 8000 compounds widely found not only in several plants, but also in aliments such as red wine, vegetables, and beer. Flavonoid’s effect against cardiovascular diseases, cancer, oxidative stress, and anti-inflammatory action is well known. Among them, quercetin (**62**) is considered a strong antioxidant thanks to its scavenging action principally ascribed both to the catechol group and OH group at the C3 position.

Starting from the 1990s, several studies have evaluated the antinociceptive effect of quercetin (**62**) [159,160,161]. Filho et al. first investigated i.p. quercetin’s antinociceptive mechanism and, interestingly, they found a significant effect in capsaicin, glutamate, and in the second phase of the formalin test, but not in the hot-plate test, suggesting that the opioid system is not involved in quercetin’s analgesic action. Naloxone pre-treatment did not revert its antinociceptive effect, while the pre-treatment with methysergide and ketanserine reverted its action, indicating a serotonin system involvement [162]. During the following years, several studies confirmed quercetin’s analgesic action in chronic pain models, including diabetic-, CCI-, cancer- and chemotherapy-induced neuropathic pain [163,164]. Among several mechanisms that can explain quercetin’s analgesic action, the most reported [165,166] are antioxidant and anti-inflammatory activities together with a mast cell membrane stabilization and PKCε-dependent TRPV1 expression [167]. Ji C. et al. confirmed the quercetin anti-inflammatory action through pro-inflammatory cytokines, such as TNF-a, IL-1b, CCL-2, and MMPs, level reduction, but also inhibition of TLR and NF-kB through TAK-1, signaling pathways [168].

Muto et al. showed that the immune system, which plays a pivotal role in neuropathic pain conditions [169,170], could be another action site of quercetin (**62**). Indeed, they demonstrated that quercetin (**62**) treatment reduced GFAP levels in astrocytes of the ipsilateral L5 dorsal root ganglia SNI mice compared to control [171]. In addition, Yang et al. discovered a significant decrease of both mRNA and protein expression of the P2X_4_ receptor, upregulated and involved in the increased mechanical and thermal hyperalgesia, followed by inhibition of p38MAPK signaling, in a mouse model of diabetic neuropathic pain. Interestingly, this receptor is also highly expressed in satellite glial cells of DRGs, confirming quercetin’s action in immune cells [172]. Moreover, Fang et al. demonstrated that quercetin (**62**) treatment in spinal cord injury significantly restored M1/M2 polarization of macrophages, preventing necroptosis of oligodendrocytes and ameliorating the demyelination of spared axon [173].

## 4. Discussion and Conclusions

Over the years, interest in natural products has grown due to their diverse molecular structures and attractive pharmacological profiles. During the earlier stage of the discovery process, the main goal is generally the identification of the bioactive compounds responsible for the biological proprieties of natural sources by using different methods basing on the structural variety, stability, and quantity of the compounds. Generally, the usage of natural components is subjected to small structural modifications to develop novel analogs with improved pharmacological profiles and minor adverse effects, but numerous currently approved small-molecule drugs are derived from both unchanged and modified natural products.

In this work, we report different natural compounds characterized by various scaffolds useful for further structural modifications and medicinal chemistry optimization, and several different models of both acute and, above all, persistent pain in which the different active principles were tested. Mainly, acetic acid-induced visceral pain, inflammatory (carrageenan-induced edema, xylene-induced mouse ear edema, cotton pellet-induced mouse granuloma, formalin-, monosodium urate-, capsaicin- and CFA-induced pain) and neuropathic pain models (CCI-, STZ-, PSNL-, chemotherapy- and HIV-induced neuropathy) were used.

Regarding the biological mechanisms of natural bioactive compounds that do not involve the opioid system, we listed different pharmacological profiles that could provide an improvement in the therapeutic area of pain treatment in Table 2.

For instance, different active principles here analyzed (e.g., sinomenine, cedrol, genistein, geniposide, and saikosaponin A) have a role in pain modulation acting on the inflammatory response reducing the mRNA expression of common pro-inflammatory cytokines or through the inhibition of p-p38 MAPK and NF-kB. Moreover, some active principles (genistein, cedrol, geniposide, solasodine, (−)-cassine, salvianolic acid A, gallic acid, and (*E*)-β-caryophyllene) control ROS overproduction by restoring GSH content and GSH/GSSG ratio, improving antioxidant enzymes activities. Other analgesic mechanisms are related to the inhibition of TRPV1 and TRPA1, as observed in the activity of (−) cassine and gallic acid, and to the agonist activity of sanguinarine versus TRPA1 that determines desensitization of sensory neurons expressing TRPA1. Moreover, some natural bioactive compounds exert their effects via a range of mechanisms like HMGB1 inhibition, gap junction blockade, and α2A-adrenoceptor antagonism. Examples of this are glycyrrhizin and other licorice-derived compounds. Moreover, some of the reported compounds promote analgesic effects by decreasing COX-2 and iNOS synthetase expression, such as leucodin and kirenol.

Overall, natural products can be considered important tools for the development or optimization of novel therapeutics. The bioactive principles derived from natural sources represent potential alternatives to conventional pain therapies. Nevertheless, additional studies are necessary to better investigate the analgesic effects of the bioactive natural principles in human pathological conditions.

## Figures and Tables

**Table 1:** Natural plant sources, active principles, and references of compounds described in the review.

Natural Source		Active Principles	Paragraph	References
Species	Family			
*Aquilaria sinensis* (Lour.) Spreng. *Aquilaria malaccensis* Lam.	Thymelaeaceae	Neopetasane (eremophilane), β-agarofuran, (−)-guaia-1(10),11-dien-15-al; 2-(2-phenylethyl)chromone; mangiferin; iriflophenone 3,5-*C*-β-diglucoside, genkwanin 5-*O*-β-primeveroside; stigmasterol, 3β-friedelanol; 4-hydroxybenzoic acid, syringic acid, isovanillic acid	3.18.	[18,19,20,21,22,23,24,25,26]
*Albizia anthelmintica* Brongn.	Fabaceae	Quercetin, kaempferol and their glucoside derivates; eucomic acid	3.29.	[27,28]
*Aquilaria crassna* Pierre *Cannabis sativa* L.	Thymelaeaceae Cannabaceae	(*E*)-β-Caryophyllene	3.20.	[29,30,31,32,33,34,35,36,37]
*Artemisia annua* L. *Artemisia californica* Less.	Asteraceae	Leucodin, α-santonin	3.19.	[38,39,40,41]
*Bauhinia glauca* ssp. *hupehana* (Craib) T.C. Chen	Leguminosae	Fisetin	3.27.	[42,43]
*Bupleurum chinense* DC.	Apiaceae	Saikosaponin A	3.25.	[44,45,46]
*Cassia leptophylla* Vogel	Fabaceae	(−)-Spectaline	3.26.	[47,48]
*Citrus sinensis* (L.) Osbeck *Citrus paradisi* Macfad *Baccharis dracunculifolia* DC.	Rutaceae Asteraceae	Isosakuranetin	3.14.	[49,50,51]
*Coffea arabica* L. *Coffea canephora* Pierre ex A. Froehner	Rubiaceae	5-Caffeoylquinic acid (5-CQA)	3.1.	[52,53,54,55,56,57,58,59,60]
*Cornus officinalis* Torr. ex Dur. *Eucalyptus globulus* Labill. *Quercus infectoria* G. Oliver *Rheum officinale* Baill. *Rheum palmatum* L.	Cornaceae Myrtaceae Fagaceae Polygonaceae	Gallic acid	3.13.	[61,62,63,64,65,66,67,68,69]
*Datura stramonium* L.	Solanaceae	Daturalactone, 12-Deoxywithastramonolide, daturilin	3.16.	[70,71,72]
*Eremocarpus setigerus* (Hook.) Benth. *Dodonaea viscosa* Jacq.	Euphorbiaceae Sapindaceae	Hautriwaic acid	3.9.	[73,74]
*Gardenia jasminoides* J. Ellis *Crocus sativus* L.	Rubiaceae Iridaceae	Crocin	3.21.	[75,76,77,78,79,80,81,82,83]
*Gardenia jasminoides* J. Ellis	Rubiaceae	Geniposide	3.23.	[84,85,86]
*Genista tinctoria* L.	Fabaceae	Genistein	3.5.	[87,88,89,90,91]
*Glycyrrhiza glabra* L. *Glycyrrhiza uralensis* Fisch. ex DC. *Glycyrrhiza inflata* Batalin	Fabaceae	Glycyrrhizin and its derivatives, carbenoxolone; licochalcone A, isoliquiritigenin, isoliquiritin	3.17.	[92,93,94,95,96,97,98,99,100,101,102,103,104,105,106,107,108,109,110]
*Hyptis emoryi* Torr. *Uapaca staudtii* Pax	Lamiaceae Phyllanthaceae	Betulinic acid	3.28.	[111,112]
*Incarvillea sinensis* Lam.	Bignoniaceae	Incarvillateine	3.30.	[113,114,115,116,117,118,119,120]
*Juniperus communis* L. *Zingiber officinale* Roscoe	Cupressaceae Zingiberaceae	Cedrol	3.4.	[121,122,123,124,125]
*Macleaya cordata* (Willd.) R.Br. *Sanguinaria canadensis* L. *Argemone mexicana* L. *Fumaria officinalis* L.	Papaveraceae	Sanguinarine	3.7.	[126,127,128,129,130]
*Polygala sabulosa* A. W. Bennett	Polygalaceae	Scopoletin, spinasterol	3.24.	[131,132]
*Populus* × *canadensis* Moench	Salicaceae	Caffeic acid phenethyl ester	3.11.	[133,134,135]
*Pueraria lobata* (Willd.) Ohwi.	Fabaceae	Puerarin	3.2.	[136,137,138,139,140,141,142,143,144,145,146,147]
*Salvia lachnostachys* Benth.	Lamiaceae	Fruticuline A	3.12.	[148,149,150]
*Salvia miltiorrhiza* Bunge, *Agrimonia pilosa* Ledeb.	Labiatae Rosaceae	Tanshinones: cryptotanshinone, 15,16-dihydrotanshinone I, miltirone, tanshinone I, tanshinone II A. Phenolic acids: salvianolic acid A and B	3.10.	[73,74,91,126,127,128,129,130,151,152,153,154,155,156,157,158]
*Salvia officinalis L.*	Lamiaceae	Quercetin	3.31.	[159,160,161,162,163,164,165,166,167,168,169,170,171,172,173]
*Senna spectabilis* (DC.) Irwin & Barneby	Fabaceae	(−)-Cassine	3.8.	[156,157]
*Sideritis bilgeriana* P.H. Davis	Lamiaceae	Chlorogenic acid	3.15.	[174]
*Siegesbeckia orientalis* L.	Asteraceae	Kirenol	3.22.	[175,176]
*Sinomenium acutum* Rehder & E.H. Wilson	Menispermaceae	Sinomenine *N*-Demethylsinomenine	3.3.	[177,178,179,180,181,182,183,184,185,186,187]
*Solanum virginianum* L.	Solanaceae	Solasodine	3.6.	[151,152,153]

**Table 2:** Active principles, chemical class, and mechanisms of action of compounds described in the review.

Active Principles	Chemical Class of Compounds	Mechanisms of Action
Neopetasane (eremophilane), β-agarofuran, (−)-guaia-1(10),11-dien-15-al; 2-(2-phenylethyl)chromone; mangiferin; iriflophenone 3,5-*C*-β-diglucoside, genkwanin 5-*O*-β-primeveroside; stigmasterol, 3β-friedelanol; 4-hydroxybenzoic acid, syringic acid, isovanillic acid	Sesquiterpenes, Chromone, Xanthone, Polyphenols, Sterols, Phenols	Inhibition of NO and pro-inflammatory cytokines.
Quercetin, kaempferol and their glucoside derivates; eucomic acid	Polyphenols Phenolic compounds	Inhibition of key inflammation enzymes like 5-LOX, COX-1, and COX-2.
(*E*)-β-Caryophyllene	Sesquiterpene	Reduction of pro-inflammatory cytokine and ROS overproduction. Decrease of COX-2 and iNOS expression, suppressed NF-κB activation.
Leucodin, α-santonin	Sesquiterpene lactones	Inhibition of COX-2 and inducible NO synthase.
Fisetin	Polyphenol	Reduction of ROS overproduction. Inhibition of MAO-A activity, and activation of 5-HT_7_ receptors.
Saikosaponin A	Triterpenoid saponin	Reduction of pro-inflammatory cytokines and decrease of the expression of p-p38 MAPK and NF-kB.
(−)-Spectaline	Piperidine alkaloid	Inhibition of TRPV1 and of excitatory amino acid, glutamate, acting through *N*-methyl-D-aspartate (NMDA) receptors.
Isosakuranetin	Polyphenol	Inhibition of transient receptor potential melastatin 3 (TRPM3).
5-Caffeoylquinic acid (5-CQA)	Polyphenol	Inhibition of NO and pro-inflammatory cytokines. Control of ROS overproduction. Decrease of neuron excitability through the enhancement of K-selective voltage-gated channels (Kv) activities.
Gallic acid	Polyphenol	Inhibition of histamine release, oxidative stress, and induction of free radical scavenging action. Reduction of pro-inflammatory cytokines and decrease of the expression of NF-kB. Inhibition of TRPA1.
Daturalactone, 12-deoxywithastramonolide, daturilin	Steroidal lactones	Inhibition of NO and pro-inflammatory cytokines.
Hautriwaic acid	Diterpene	Reduction of pro-inflammatory cytokines and enhancement of IL-10 activity.
Crocin	Carotenoid glycoside	Antioxidant properties through modulation of GPx, GST, CAT, and SOD.
Geniposide	Iridoid glycoside	Reduction of pro-inflammatory cytokine and ROS overproduction. Activation of spinal GLP-1Rs.
Genistein	Isoflavone	Reduction of pro-inflammatory cytokines and ROS overproduction. Block of the activity of human Ca_v_3.3 channel.
Glycyrrhizin and its derivatives, carbenoxolone; licochalcone A, isoliquiritigenin, isoliquiritin	Triterpenoid saponins, polyphenols	Reduction of pro-inflammatory cytokines. HMGB1 inhibition, gap junction blockade and α2_A_-adrenoceptor antagonist profile.
Betulinic acid	Pentacyclic triterpenoid	Antinociceptive action through interaction with Cav3.2 (T-type) and Cav2.2 (N-type).
Incarvillateine	Monoterpene alkaloid	Antinociceptive action through adenosine receptors’ agonist action.
Cedrol	Sesquiterpene	Reduction of pro-inflammatory cytokines and ROS overproduction. Inhibition of TRPA1.
Sanguinarine	Benzyl isoquinoline alkaloid	Reduction of pro-inflammatory cytokine. Selective agonist of TRPA1 channel acting through desensitization of sensory neurons expressing TRPA1.
Scopoletin, spinasterol	Coumarin, steroid	Glutamatergic transmission inhibition. Reduction of pro-inflammatory cytokine.
Caffeic acid phenethyl ester	Polyphenol	Reduction of pro-inflammatory cytokines and decrease of the expression of p-p38 MAPK and NF-kB.
Puerarin	Isoflavone glycoside	Decrease of P2X3 nociceptive transmission and Na_v_ channels blockade. Inhibition of TRPV1.
Fruticuline A	Diterpene	Inhibition of TNF activation.
Tanshinones: cryptotanshinone, 15,16-dihydrotanshinone I, miltirone, tanshinone I, tanshinone II A. Phenolic acids: salvianolic acid A and B	Diterpenes, polyphenols	Reduction of pro-inflammatory cytokines and decrease of the expression of p-p38, MAPK, and NF-kB. Control of ROS overproduction. Reduction of NO release, attenuation of COX-1, COX-2.
Quercetin	Polyphenol	Reduction of pro-inflammatory cytokines and decrease of the expression of NF-kB.
(−)-Cassine	Piperidine alkaloid	Reduction of ROS overproduction, Inhibition of TRPV1 and TRPA1. Down-regulation of COX-2, MAP/ERK pathway, and NF-κB expression.
Chlorogenic acid	Polyphenol	Reduction of pro-inflammatory cytokines and decrease of the expression of NF-kB.
Kirenol	Diterpenoid	Inhibition of COX-2 and inducible NO Synthase.
Sinomenine *N*-demethylsinomenine	Alkaloids	Reduction of pro-inflammatory cytokines. Reduction of cellular excitability via voltage-gated sodium channels.
Solasodine	Steroidal glycoalkaloid	Reduction of pro-inflammatory cytokine and ROS overproduction.

## Abbreviations

5-HTP, 5-hydroxytryptophan; AGE, advanced glycation end-product; BBB, blood–brain barrier; BDNF, brain-derived neurotrophic factor; CaMKII, calcium–calmodulin-dependent protein kinase II; CAT, catalase; CB1R, cannabinoid type 1 receptors; CB2R, cannabinoid type 2 receptor; CCI, chronic constriction injury; CFA, complete Freund’s adjuvant; COX-1, cyclooxygenase-1; COX-2, cyclooxygenase-2; CREB, cAMP response element-binding; CRP, C reactive protein; CXCR4, C-X-C motif chemokine receptor 4; DRG, dorsal root ganglion; EGFR, epidermal growth factor receptor; eNOS, endothelial nitric oxide synthase; ER, estrogen receptor; ERK, extracellular signal-regulated kinases; FDA, food and Drug Administration; FST, forced swim test; GABA_A_, gamma amino butyric acid-A; GAP-43, growth associated protein-43; GFAP, glial fibrillary acidic protein; GLP-1R, glucagon-like peptide-1 receptors; GPx, Glutathione peroxidase; GSH, glutathione; GSSG, glutathione disulfide; GST, glutathione S-transferase; HEK29,3 human embryonic kidney 293; HMGB1 high mobility group box 1; HPLC high-performance liquid chromatography; i.p., intraperitoneal; i.t., intrathecal; ICR, Institute of Cancer Research; IL-1β, interleukin-β; iNOS, inducible inhibitor nitric oxide synthase; ITS, internal transcribed spacer; JAK3, Janus kinase 3; JNK, c-Jun N-terminal kinases; Lox-1, lectin-type oxidized LDL receptor 1; LPS, lipopolysaccharide; LTB4, leukotriene B4; MAO, monoamine oxidase; MAPK, mitogen activated protein kinase; MBP, myelin basic protein; MCP-1, monocyte chemoattractant protein-1; MDA, malondialdehyde; MPO, myeloperoxidase; mRNA, messenger ribonucleic acid; NF-κB, nuclear factor κB; NK, tachykinin; NLRP3, NLR family pyrin domain containing 3; NO, nitric oxide; OX42, integrin αM/CD11b antibody; p.o., oral; PGE2, prostaglandin E2; PI3K/Akt, phosphatidylinositol 3’-kinase/protein kinase B; PPAR-γ, peroxisome proliferator-activated receptor gamma; PSNL, partial sciatic nerve ligation; PWT, paw withdrawal threshold; ROS, reactive oxygen species; s.c., subcutaneous; SCI, spinal cord injury; Smad, suppressor of mothers against decapentaplegic; SNI, spared nerve injury; SNL, spinal nerve ligation; SOD, superoxide dismutase; STZ, streptozotocin; TLR, toll-like receptor; TLR4, Toll-like receptor 4.TNF, tumor necrosis factor; TRPA1, transient receptor potential cation channel, member A1; TRPV1, transient receptor potential vanilloid 1; TTX-R, tetrodotoxin-resistant; TTX-S, tetrodotoxin-sensitive; vWF, von Willebrand factor.
